# Effects of operational parameters on bacterial communities in Hong Kong and global wastewater treatment plants

**DOI:** 10.1128/msystems.01333-23

**Published:** 2024-02-27

**Authors:** Yulin Zhang, Yu Deng, Chunxiao Wang, Shuxian Li, Frankie T. K. Lau, Jizhong Zhou, Tong Zhang

**Affiliations:** 1 Environmental Microbiome Engineering and Biotechnology Lab, Department of Civil Engineering, The University of Hong Kong, Hong Kong, China; 2 Drainage Services Department, The Government of the Hong Kong Special Administrative Region of the People’s Republic of China, Wanchai, Hong Kong, China; 3 Institute for Environmental Genomics, Department of Microbiology and Plant Biology, and School of Civil Engineering and Environmental Sciences, University of Oklahoma, Norman, Oklahoma, USA; 4 Macau Institute for Applied Research in Medicine and Health, Macau University of Science and Technology, Macau, China; University of Pretoria, Pretoria, South Africa

**Keywords:** wastewater treatment plants, activated sludge, nitrification, assembly mechanism, operational parameters, microbial dark matter

## Abstract

Wastewater treatment plants (WWTPs) are indispensable biotechnology facilities for modern cities and play an essential role in modern urban infrastructure by employing microorganisms to remove pollutants in wastewater, thus protecting public health and the environment. This study conducted a 13-month bacterial community survey of six full-scale WWTPs in Hong Kong with samples of influent, activated sludge (AS), and effluent to explore their synchronism and asynchronism of bacterial community. Besides, we compared AS results of six Hong Kong WWTPs with data from 1,186 AS amplicon data in 269 global WWTPs and a 9-year metagenomic sequencing survey of a Hong Kong WWTP. Our results showed the compositions of bacterial communities varied and the bacterial community structure of AS had obvious differences across Hong Kong WWTPs. The co-occurrence analysis identified 40 pairs of relationships that existed among Hong Kong WWTPs to show solid associations between two species and stochastic processes took large proportions for the bacterial community assembly of six WWTPs. The abundance and distribution of the functional bacteria in worldwide and Hong Kong WWTPs were examined and compared, and we found that ammonia-oxidizing bacteria had more diversity than nitrite-oxidizing bacteria. Besides, Hong Kong WWTPs could make great contributions to the genome mining of microbial dark matter in the global “wanted list.” Operational parameters had important effects on OTUs’ abundance, such as the temperature to the genera of *Tetrasphaera*, *Gordoni*a and *Nitrospira*. All these results obtained from this study can deepen our understanding of the microbial ecology in WWTPs and provide foundations for further studies.

**IMPORTANCE:**

Wastewater treatment plants (WWTPs) are an indispensable component of modern cities, as they can remove pollutants in wastewater to prevent anthropogenic activities. Activated sludge (AS) is a fundamental wastewater treatment process and it harbors a highly complex microbial community that forms the main components and contains functional groups. Unveiling “who is there” is a long-term goal of the research on AS microbiology. High-throughput sequencing provides insights into the inventory diversity of microbial communities to an unprecedented level of detail. At present, the analysis of communities in WWTPs usually comes from a specific WWTP and lacks comparisons and verification among different WWTPs. The wide-scale and long-term sampling project and research in this study could help us evaluate the AS community more accurately to find the similarities and different results for different WWTPs in Hong Kong and other regions of the world.

## INTRODUCTION

Wastewater treatment plants (WWTPs) are an indispensable component of modern cities, as they can remove pollutants in wastewater to prevent anthropogenic activities (1). Activated sludge (AS) (2) is a fundamental wastewater treatment process and it harbors a highly complex microbial community that forms the main components and contains functional groups. Unveiling “who is there” is a long-term goal of the research on AS microbiology. Currently, high-throughput sequencing (HTS) provides insights into the inventory diversity of microbial communities to an unprecedented level of detail and enables the detection of more taxa including a large percentage of uncultured organisms.

Studies of compositions of microbial communities have increased researchers’ understanding of the associations and relationships between microorganisms in both natural and engineering systems (3, 4). For example, seasonal variation had a big impact on the AS bacterial community (1, 2) and the biodegradability of the influent could determine WWTP performance by affecting the microbial community of AS (5). Industrial AS exhibits a unique profile that is distinct from the characteristics of municipal AS due to the composition difference between industrial and municipal wastewater (6). Apart from the overall microbial communities, the co-occurrence patterns of microbial communities will provide new insights into the AS system (1). Network analysis is a useful tool for studying microbial associations and has been applied to determine microbial coexistence in complex communities, such as soils (7) and oceans (8). The application of such network analysis on the AS community may improve our understanding of potential microbial relationships for bacterial adjustment (9) and provide ideas to improve the treatment efficiency of AS (2, 10, 11). Understanding the assembly rules like neutral and niche theories (12) and finding corresponding assembly driving factors of the microbial community are also important ecological problems. Both stochastic and deterministic processes operate concurrently for WWTPs (13). For instance, researchers revealed that microbial communities in AS were dominated by deterministic processes with temperature as the most important driver (14, 15). However, a global AS survey (16) and an AS study in China (17) showed more crucial positions of stochastic processes in community assembly (18).

One of the most important functions of WWTPs is to remove nutrients such as nitrogen and phosphorus from sewage (1). These important processes are performed by functional groups such as ammonia-oxidizing bacteria (AOB), nitrite-oxidizing bacteria (NOB) and polyphosphate-accumulating organisms (PAOs) (19), and can be affected by bulking and foaming bacteria (BFB). A comprehensive understanding of functional groups will provide valuable insight into the performance evaluation and routine maintenance of WWTPs. Genome sequencing enhances our understanding of the biological world by providing blueprints for the evolutionary and functional diversity that shapes the biosphere (20). However, microbial genomes that are currently available are of limited phylogenetic breadth due to our inability to culture many environmental microorganisms (21). These unknown microbes whose genomes remain uncharacterized are colloquially called “microbial dark matter” (MDM) (22). The proportions of MDM in WWTPs are of great value to define their roles and a study in 2023 conducted an accurate survey of the MDM conditions in global WWTPs to propose a “wanted list” (23). We should evaluate the MDM conditions of Hong Kong WWTPs and explore their potential contributions to the MDM exploration for global WWTPs.

AS is an engineered ecosystem whose microbial communities originate from inoculation sludge that is purposefully chosen and could be affected by various factors, such as geographical locations, seasonal (environmental) variations (24), influent wastewater characteristics (25) and operational parameters (26). The rapid increase in both the quantity and complexity of daily operational data in the field of environmental engineering demands advanced data analysis approaches like redundancy analysis (RDA) to find or predict key parameters for bacteria (27). However, most earlier studies evaluated the effects of parameters on the whole AS microbiota and did not provide feedback for specific genera.

At present, the analysis of communities in WWTPs usually comes from a specific WWTP and lacks comparisons and verification among different WWTPs. The wide-scale and long-term sampling project could help us evaluate the AS community more accurately to find synchronous and asynchronous results for different WWTPs in Hong Kong and other regions of the world. In this research, samples (influent, AS, and effluent) were monthly collected from six full-scale WWTPs in Hong Kong for 13 months to conduct the 16S rRNA gene amplicon sequencing, and Hong Kong AS data were compared with worldwide WWTP results to address the following ecological questions of the bacterial community in AS: (i) bacterial community conditions for six Hong Kong WWTPs, such as compositions, co-occurrence patterns and assembly mechanisms; (ii) abundance and distribution of functional groups in Hong Kong and global WWTPs; (iii) MDM conditions in Hong Kong and global WWTPs; and (iv) effects of operational parameters on OTUs’ abundance in Hong Kong and global WWTPs. This investigation will advance our knowledge of the microbial ecology in AS systems to lay the groundwork for future investigations into the community regulation approach for WWTPs.

## MATERIALS AND METHODS

### WWTP, sampling, and sample pretreatment information

The influent (IN), AS, and effluent (EFF) samples were collected from six secondary WWTPs that applied biological treatment process in Hong Kong, including Shatin (ST), Tai Po (TP), Shek Wu Hui (SWH), Yuen Long (YL), Sai Kung (SK), and Stanley (STL) WWTP. All these WWTPs adopted the conventional AS process for carbon and nitrogen removal except for STL WWTP, which also applies the integrated fixed film activated sludge process (Table S1). Among the six WWTPs, ST WWTP is the largest WWTP in Hong Kong treating 260,000 m^3^ of sewage from over 650,000 citizens per day. Besides, ST WWTP and TP WWTP treat sewage with a salinity of ~1% since Hong Kong uses seawater for toilet flushing with stable performance. Although the compositions of influent vary among WWTPs, the performances of the six WWTPs were generally stable throughout the year and the effluent of all WWTPs always meets the standard according to the record of Hong Kong DSD. Their technical and operational details are shown in Tables S1 and S2, respectively.

Individual samples were taken monthly from January 2018 to January 2019 in WWTPs and finally, we got 74 influent, 74 AS, and 74 effluent samples since the sampling work of SK WWTP was suspended following the damage caused by a super typhoon in September 2018. An amount of 300 mL of influent (mixing with 300 mL of 100% ethanol on site), 25 mL of AS (mixing with 25 mL of 100% ethanol on site), and 5 L of effluent (from the final sedimentation tank) was taken on-site in capped containers and transported to the laboratory immediately. Then, 600 mL of influent samples was centrifuged at 4750 rpm for 10 min (Beckman Coulter Avant J-15R, USA) to remove the supernatant and keep concentrated microorganisms, and then the remaining samples were further centrifuged at 15,000 × *g* for 3 min (Beckman Coulter Microfuge 20R, USA) to collect pellets for DNA extraction. An amount of 2 mL of AS samples was centrifuged at 15,000 ×*g* for 3 min (Beckman Coulter Microfuge 20R, USA) to collect pellets for DNA extraction. 5 L of effluent samples was filtered with a 0.45-µm cellulose acetate membrane (Advantec MFS, Inc., USA) to collect the biomass for DNA extraction.

### DNA extraction, amplicon sequencing, and data processing

The genomic DNA was extracted by the FastDNA SPIN Kit for Soil (MPBiomedicals, France) according to the protocol of the manufacturer. The quality and quantity of extracted DNA were determined by NanoDrop Spectrophotometer ND-100 (ThermoFisher Scientific, USA) and Qubit 2.0 fluorometer (Life Technologies, USA), respectively. 300 bp paired-end sequencing reads were generated on a HiSeq (Illumina, San Diego, CA, USA) using the V3-V4 PCR primer set of 341F ACTCCTACGGGAGGCAGCAG and 806R GGACTACHVGGGTWTCTAAT by Beijing Genomics Institute (BGI, China). The raw data of 16S rRNA reads have been submitted to the National Center for Biotechnology Information (NCBI) with the BioProject ID PRJNA1012295.

DADA2 (28) in QIIME2 (version 2020.2) (29) was used to correct DNA sequences by establishing a customized sequence error mode based on the input sequences after the sequence quality control. The singletons were removed to guarantee sequence quality, and then the sequence number was normalized to 25,000 (Fig. S1) followed by the OTU generation with vsearch (30) at a 97% similarity level. The Silva 138 database (31) was applied to annotate the taxonomy information and the functional groups such as AOB, NOB, PAO and BFB groups were extracted by taxonomy annotation according to Table S3.

### Basic information and data analysis of global WWTPs

The worldwide WWTP survey contained 1,186 AS samples from 269 WWTPs. The information details of worldwide WWTPs and generation steps of OTUs were described in the previous study (16). The representative sequences were aligned by MAFFT (version 7.505) (32) for constructing the phylogenetic tree with FastTree2 (version 2.1.10) (33) and visualizing by iTOL (version 6) (34). The functional groups of global WWTPs were extracted by taxonomy annotation with the Silva 138 database (31) according to Table S3. AOB and NOB OTUs of Hong Kong and global WWTPs were aligned with the Genome Taxonomy Database (GTDB R207) (35) by BLASTn (36) with the top-hit result to determine the genome information by a similarity cutoff of 97%.

### Neutral community model for communities

The relationship between OTU detection frequency and their relative abundance within the community was predicted using the neutral community model (NCM) in R (version 4.3.1), which was used to assess the potential significance of stochastic processes on community assembly. The model employed in this study followed the methodology developed by Chen et al. (37) and enabled scholars to assess the crucial ecological processes that were challenging to detect directly but had a significant impact on the microbial community, such as dispersal and drift. In the analysis, *Nm* determined the correlation between occurrence frequency and regional relative abundance and *R*^2^ represented the overall fit to the neutral model. Using 1,000 bootstrap replicates, the 95% confidence intervals around all fitting statistics were calculated.

### Co-occurrence network creation and analysis

The co-occurrence of species-species associations was analyzed with the calculation of all pair-wise Spearman’s rank coefficients (*P* < 0.05) among OTUs that had >0.01% average relative abundance in samples and visualized with Gephi (version 0.9) (38). Meanwhile, networks were also constructed by the spiec.easi() function from the R package of SpiecEasi (version 1.1.1) (39) with the default settings of the “mb” parameter to estimate the conditional dependence of each pair of OTUs. Relationships of species identified by both the Spearman (*P* < 0.01) and SpiecEasi were conserved for the following co-occurrence analysis among different WWTPs in Hong Kong. The core (showed in >80% of samples with a relative abundance >0.1%) and functional OTUs were used to further filter species relationships. Finally, 40 pairs of relationships were obtained (Fig. 2; Table S4) and visualized with Gephi (version 0.9) (38).

### MDM conditions, operational parameters and long-term data of Hong Kong WWTPs

The proportions of the genome-sequenced cells and taxa were calculated according to the method described in the published research (23). *P*_number_ and *P*_abundance_ were used to represent the genome-sequenced proportions of taxa (total OTU number) and corresponding cell number (total relative abundance sum). In summary, sequences of Hong Kong WWTPs were aligned as query via BLASTn (E value <1e^–5^) (36) with genomes in GTDB R207 to pick the top-hit sequence meeting the standard of 100% alignment and 100% identity and calculate the *P*_number_ and *P*_abundance_. To examine the distribution of the global “wanted list” in Hong Kong, sequences of Hong Kong WWTPs were aligned as query via BLASTn (E value <1e^–5^) (36) with sequences in the global “wanted list” (23) to pick the top-hit sequence meeting the standard of 100% alignment and 100% identity. The operational parameters of WWTPs were provided by Hong Kong DSD, and RDA was conducted by R (version 4.3.1). The long-term metagenomic sequencing data of ST AS (over 9 years) was collected from the former research (40) to extract the wanted metagenome-assembled genomes (MAGs) according to their taxonomic annotations.

## RESULTS AND DISCUSSION

### The conditions of the bacterial community for Hong Kong WWTPs

#### The composition, diversity and dynamics of the bacterial community for Hong Kong WWTPs

**Fig 1:**
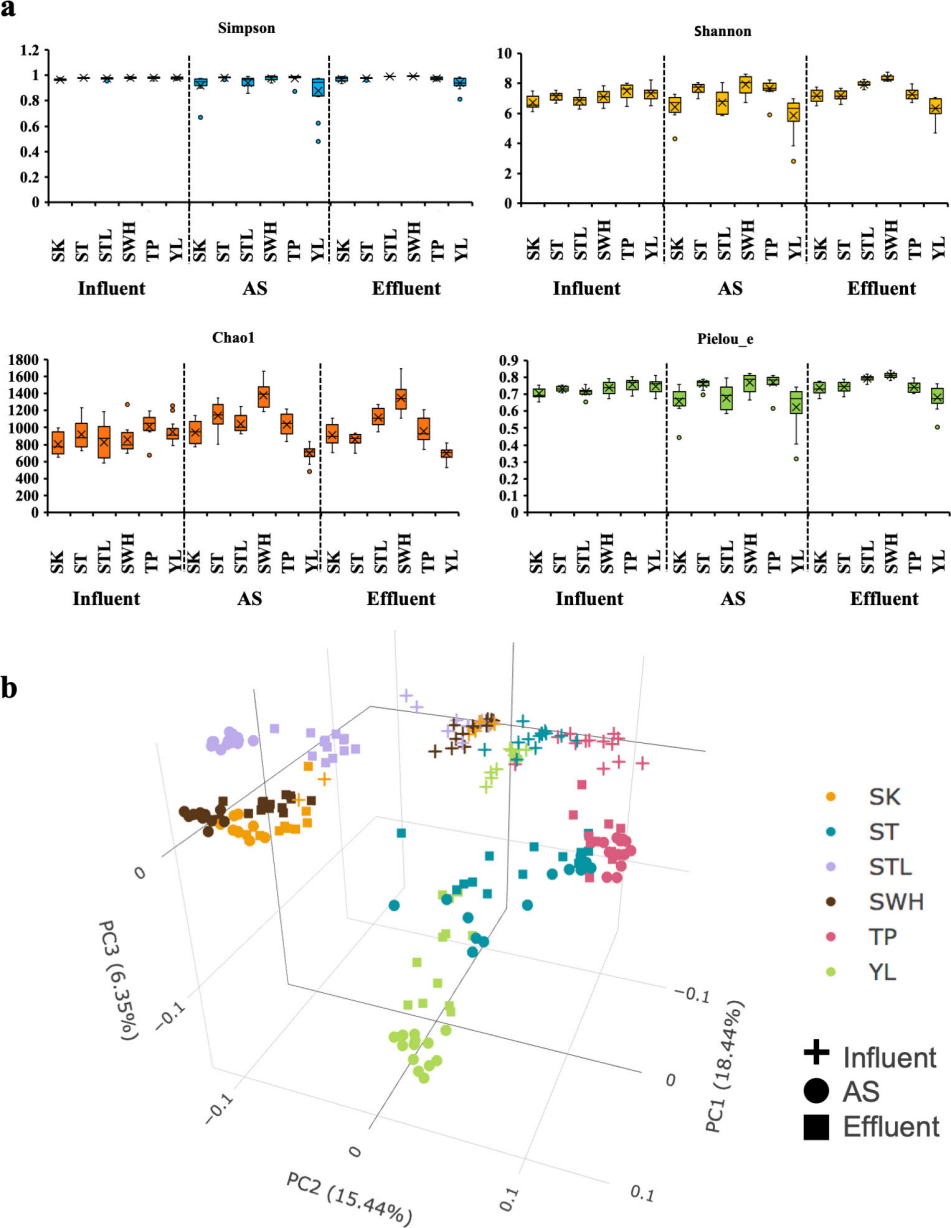
Diversity analysis of six Hong Kong WWTPs. (**a**) The box chart of alpha diversity of influent, AS, and effluent. Outliers were marked. (**b**) The PCoA plot of influent, AS and effluent. The analysis was conducted by comparing pairwise microbial community dissimilarity distances using the Bray-Curtis algorithm. Each dot represented the microbial community structure of an individual sample. The colors and shapes of the dots represented WWTPs and sample types, respectively.

In total, AS samples of six Hong Kong WWTPs generated 7,649 OTUs with 48 phyla of bacteria (7,569 OTUs), 3 phyla of archaea (10 OTUs), and 70 OTUs with unclassified bacterial phyla (Fig. S2). Consistent with previous studies (1, 2), the phylum abundance of communities varied in different WWTPs but the dominant phyla in AS were the same, which were *Proteobacteria*, *Bacteroidetes*, *Chloroflexi*, *Patescibacteria*, *Actinobacteria*, *Acidobacteria,* and *Nitrospirae*. The performance of WWTPs could be more stable if AS communities have a higher diversity (5). Overall, the bacterial diversity of AS was relatively stable throughout the year, which was consistent with the stable performance of these six full-scale WWTPs (Fig. 1a; Fig. S3). The bacterial diversity of AS for different WWTPs varied greatly, and SWH and YL WWTPs had the highest and lowest richness and diversity, respectively. AS communities showed higher richness and evenness than influent and effluent communities in most WWTPs, while the diversity of effluent communities displayed notable fluctuations as they had the largest standard deviation among the three types of samples (Fig. S3).

The Bray-Curtis dissimilarity distance and principal coordinates analysis (PCoA) plot based on the relative abundance of bacteria at the phylum level was applied to calculate and distinguish the compositional differences and community relationships among WWTPs samples. Fig. 1b showed that the influent from different WWTPs had similar community compositions at the phylum level when compared with other types of samples (i.e., AS and effluent). This similarity can be attributed to the fact that the bacteria in influent mainly originated from the human gut (41, 42) and sewer pipes (43), and Hong Kong had a limited geographical area with similar dietary habits among its inhabitants. Although some WWTPs contain a portion of industrial (YL) or slaughterhouse (SWH) wastewater, the discrepancy in microbial communities caused by this part of wastewater may not be reflected at the phylum level. To a certain extent, the microbial communities identified in effluent samples bore a striking resemblance to those found in AS samples, as bacteria present in the effluent can be viewed as the unsettled portion of AS (44, 45). Except for ST WWTP which had a temporal succession (Fig. S4), Hong Kong AS had a stable community structure. The AS of SK and SWH WWTP, as well as the AS from ST (201808 to 201901 in Fig. S4) and TP WWTP, had similar bacterial communities. The AS from YL and STL WWTP had their unique cluster patterns mightily due to the significant proportion of industrial wastewater in YL WWTP and the unique integrated fixed film activated sludge process of STL WWTP.

#### Co-occurrence analysis reveals synchronized species-species associations in Hong Kong WWTPs

Network analysis showed Hong Kong WWTPs had different complexity and patterns with species in ST WWTP had the most relationships while STL WWTP had the lowest one (Fig. S5; Table S5). Similar to other studies (24), positive correlations dominated relationships among species of six WWTPs while species in ST WWTP contained many negative correlations. A module in networks is a group of OTUs that have lots of connections among themselves but very few connections with other OTUs out of that group, and bacteria in the same module might carry out similar tasks (46), which means more “small modules” could increase network stability. Hong Kong WWTPs had lots of modules in networks, which might contribute to the stable treatment effect.

**Fig 2:**
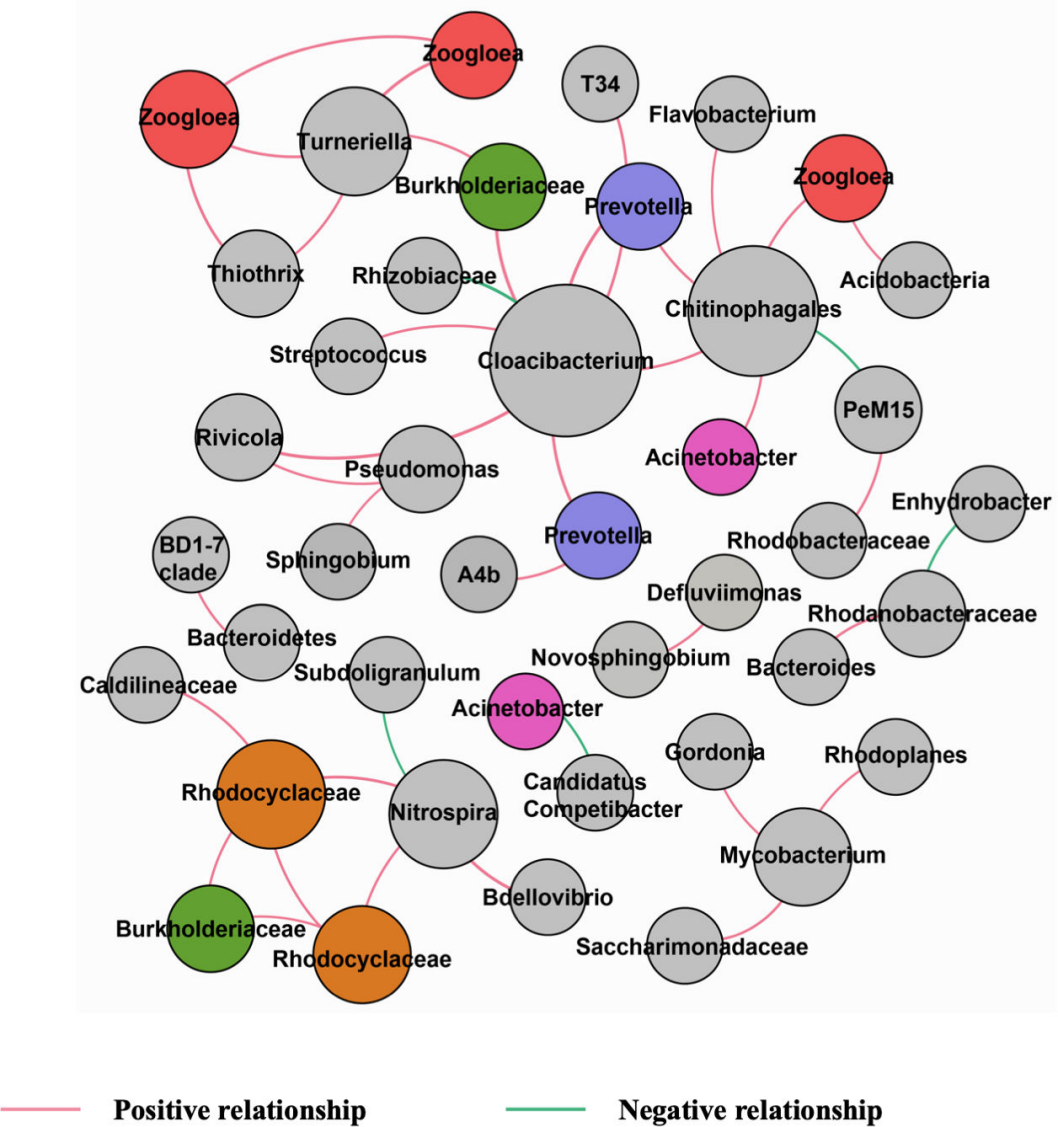
Forty pairs of co-occurrence relationships identified by the network analysis. Node size was determined by relationship frequency and OTUs that occurred repeatedly were marked with the same colors. Red and green edges represented positive and negative relationships, respectively.

In addition to species correlations of each WWTP, the relationships that exist among different WWTPs are more significant since they may represent solid and universal associations between two species that can greatly deepen the current understanding of microbial communities and contribute to the regulation of microbial communities. Fig. 2 shows 40 pairs of co-occurrence relationships identified by the network analysis. This co-occurrence pattern contained seven distinct groups and revealed common relationships between NOB/BFB and other bacteria in WWTPs. For instance, *Nitrospira* had occurrence patterns with two genera (*Bdellovibrio* and *Subdoligranulum*) and one core OTU belonging to the family *Rhodocyclaceae. Bdellovibrio* is a genus of gram-negative and obligate aerobic bacteria. One of this genus’s prominent traits is that its members may feed on their hosts’ biopolymers, such as proteins and nucleic acids, by preying upon other gram-negative bacteria (47). *Bdellovibrio* is putative predator of *Nitrospira spp*. (48, 49), while it could also prey on bacteria that suppress nitrification if a positive co-relationship exists between *Bdellovibrio* and *Nitrospira. Rhodocyclaceae* widely exists in Hong Kong WWTPs as the core bacteria with the average abundance ranging from 0.6% to 1.4%. With diverse physiological characteristics, bacteria under the *Rhodocyclaceae* family could degrade a wide range of carbon sources with electron acceptors of nitrate or nitrite and may promote nitrification to some extent. As strictly anaerobic bacteria, *Subdoligranulum* will be suppressed by high concentrations of oxygen, while nitrification could be active under such conditions.

#### Stochastic processes shape the community assembly of Hong Kong WWTPs

**Fig 3:**
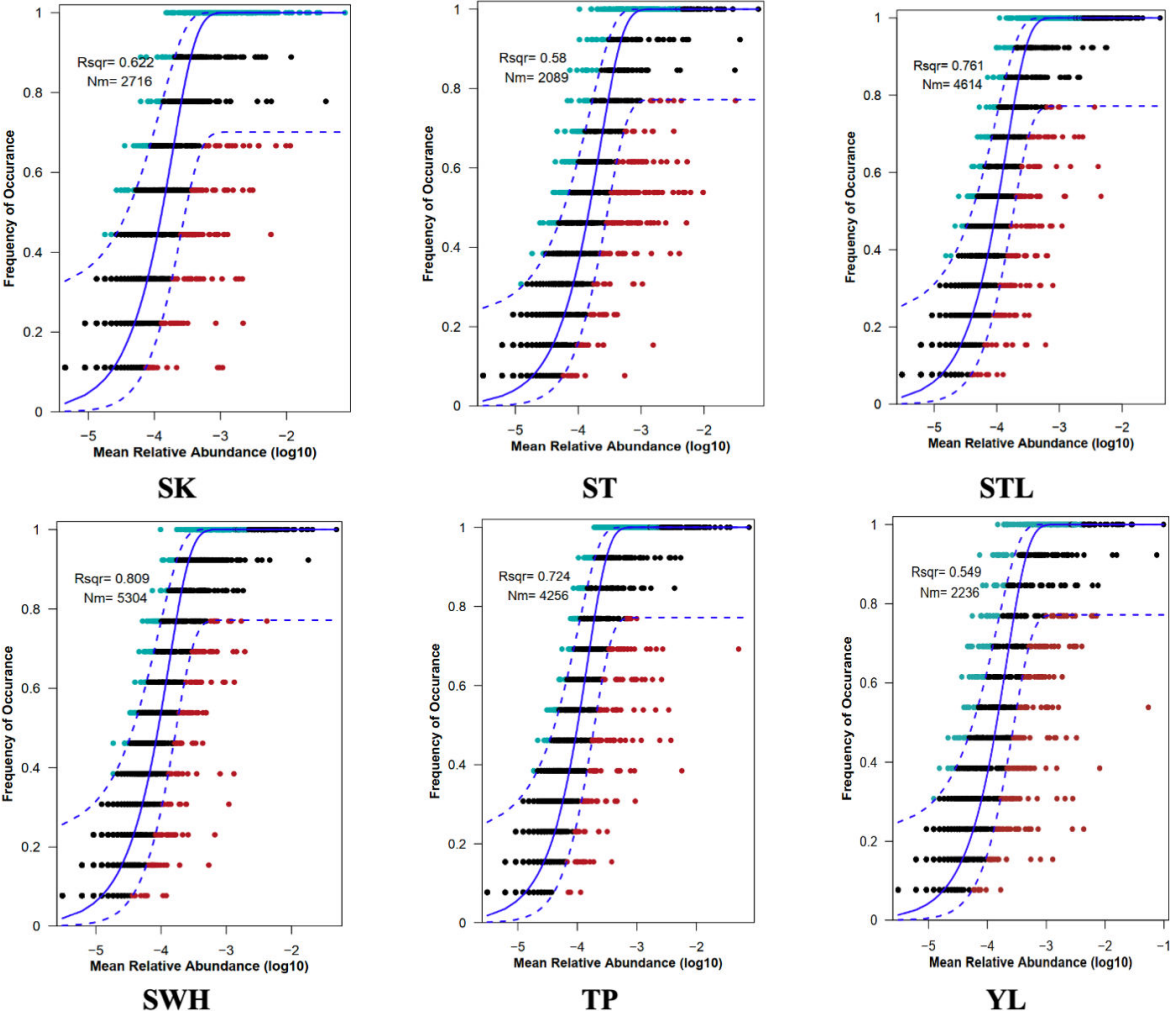
Fit of the neutral community model (NCM) of community assembly for six Hong Kong WWTPs. The solid blue lines provided the best fit to the NCM, while the dashed blue lines showed 95% confidence intervals around the model prediction. Different colors indicated OTUs that occurred more or less frequently than expected by the NCM. *Nm* represents the metacommunity size times immigration and *R^2^* was the model’s fit.

Community assembly mechanism is one of the most compelling questions in the ecological study (13, 37), especially for a community that has temporal dynamics like AS. It could elucidate driving factors that shaped the community structure and provide the theoretical basis for the regulation of AS communities (50). Stochastic and deterministic processes are two key components of assembly mechanisms. Results generated from NCM (Fig. 3) showed that all six Hong Kong WWTPs were dominated by stochastic processes (*R^2^* >0). Besides, the NCM successfully estimated a large proportion of relationships between the occurrence frequency of OTUs and their relative abundance fluctuations with the explained community variance from 54.9% (YL WWTP) to 80.9% (SWH WWTP) for six Hong Kong WWTPs. The *Nm* had the smallest and largest values in ST WWTP (*Nm* = 2,089) and SWH WWTP (*Nm* = 5,304), respectively, indicating that the frequency of species dispersal for bacteria was lower in ST WWTP while higher in SWH WWTP.

Stochastic processes dominated in communities with high diversity (51) and this stochastic dominance situation for AS was also verified in different regions (16, 24). It is reasonable that most of the OTUs in AS are dominated by stochastic processes. On the one hand, AS is an open system that receives lots of microorganisms from the influent every day and should be very vulnerable to ecological drift (52). On the other hand, functional redundancy (different populations share a similar or the same function) will increase neutrality and make functionally redundant populations more susceptible to drift from the community (13, 53). AS appears to be a microbial community that has a quite high diversity and is functionally redundant in the metabolism of lipid (40), amino acid (54), and carbohydrate metabolisms (55). Actually, previous studies often evaluated the assembly mechanism at the community level, and it would be meaningful to consider ecological processes at a lower level (e.g., the level of individual taxa or lineages) since actions of assembly processes are typically at the genotype or population level (10, 13, 18), such as the tool of Infer Community Assembly Mechanisms by Phylogenetic-bin-based null model (10). This kind of analysis may provide some novel perspectives on community assembly by finding influenced bacteria and corresponding parameters.

### The conditions of functional groups for Hong Kong and global WWTPs

#### Abundance and distribution of AOB and NOB groups in Hong Kong and global WWTPs

**Fig 4:**
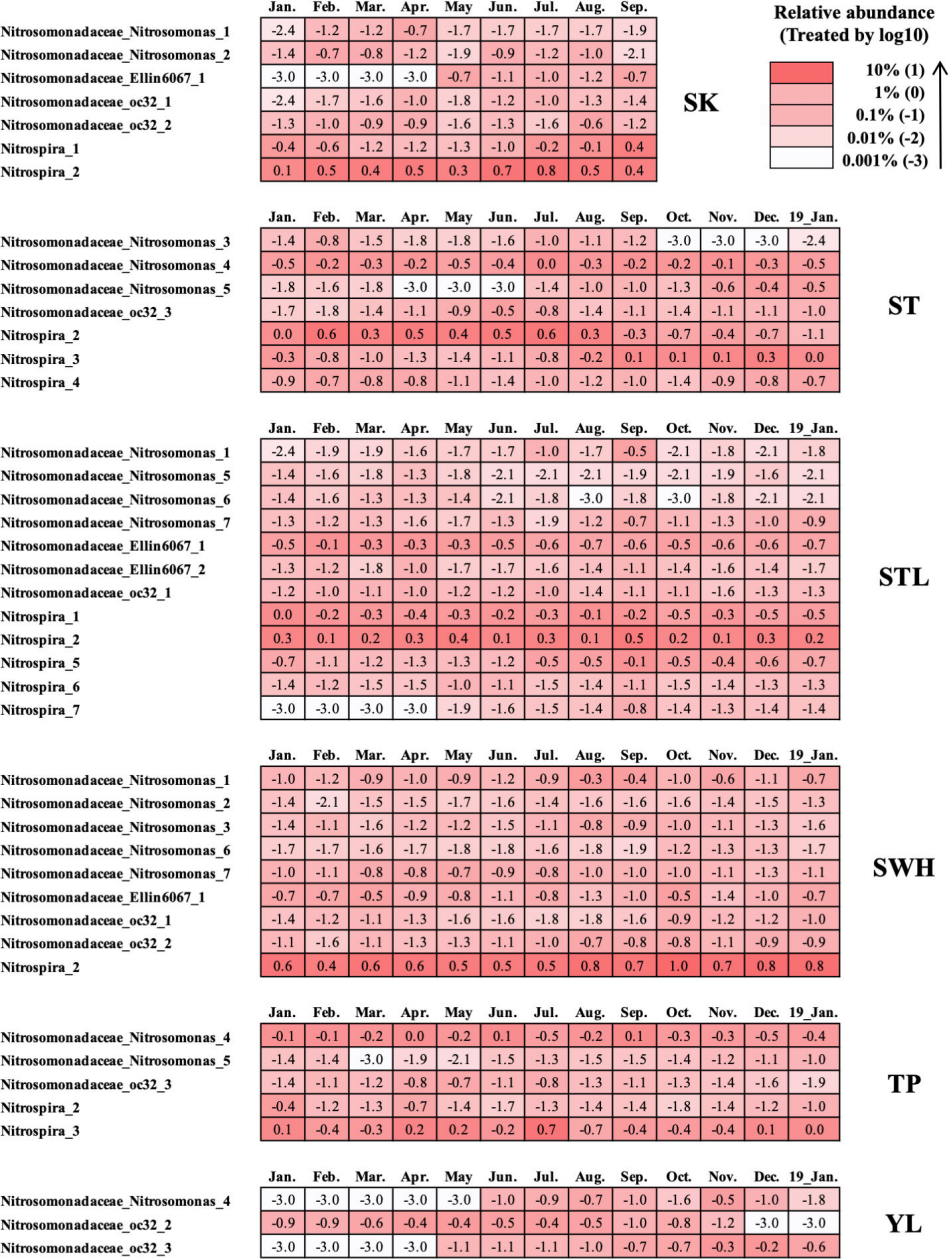
Composition, abundance, and distribution of AOB and NOB in Hong Kong WWTPs. The number in the figure represented the abundance and the abundance was treated by log10. Only the OTUs with an average abundance >0.01% were reserved. 19_Jan. represented the sample collected in January 2019.

AOB and NOB are two kinds of bacteria related to nitrogen removal (56). AOB can catalyze ammonium to nitrite and NOB could oxidize the nitrite to nitrate (57). Together, they complete the whole nitrification process. In all, 39 OTUs were defined as AOB (Fig. 4) in Hong Kong WWTPs and they all belonged to the *f_Nitrosomonadaceae* (a key member of the phylum *Proteobacteria*) with six genera: *g_Nitrosomonas*, *g_oc32*, *g_Ellin6067*, *g_DSSD61*, *g_mle1-7,* and *g_966–1*. Among them, *Nitrosomonas* was the most universal and typical AOB that appeared in all WWTPs, and *Nitrosomonadaceae_Nitrosomonas_4* in TP WWTP had the highest average abundance of 0.60%. However, the distribution of AOB across various WWTPs is highly diverse, and no unique AOB has been identified to be consistently dominant across all WWTPs. For the distribution of the AOB diversity, SWH WWTP had the most diverse AOB species across Hong Kong WWTPs in this study, although these species all had low abundance (0.07% average). However, YL WWTP had the lowest diversity with only three AOB species identified but these three AOBs had a high average abundance of 0.16%.

NOB distributes in different lineages like the genus *g_Nitrobacter* (57), *g_Nitrospina*, *g_Nitrospira*, *g_Nitrolancea*, *g_Nitrotoga,* and *g_Nitrococcus* (58), and has been detected in diverse environments such as soil (59), marine (60), WWTPs (61), and drinking water (62). The predominant NOB of different WWTPs may vary due to changes in wastewater types and operating parameters. For instance, a previous study showed that *Nitrotoga* was the primary NOB in Danish WWTPs (63). In another report, some full-scale WWTPs had a low abundance of both *Nitrotoga* and *Nitrospira* but *Nitrotoga* maintained high potential activities (64) and was recognized as the key nitrite oxidizer (61). The represented NOB in Hong Kong WWTPs was the Genus *Nitrospira* in Phylum *Nitrospirae*. In total, seven *Nitrospira* species were detected in Hong Kong WWTPs. Among them, *Nitrispira_2* was the main NOB that was widely present in all WWTPs except for YL WWTP, and its abundance was up to 4.52% average in SWH WWTP. The uniqueness of SWH WWTP in AOB and NOB conditions may be due to its content of slaughterhouse wastewater. Besides, STL WWTP had the most types of NOB with five types appearing. The average abundance ratio of AOB/NOB for WWTPs in Hong Kong (except for YL WWTP) was 0.4 with the highest ratio of 1.3 for TP WWTP and the lowest ratio of 0.1 for SK WWTP. In addition to AOB and NOB, anaerobic ammonium oxidation (anammox) bacteria can remove ammonia from wastewater by converting nitrite and ammonium ions into diatomic nitrogen and water directly (65) to contribute to the global nitrogen cycle. Interestingly, sequencing results of the 16S rRNA gene showed that anammox bacteria only presented in YL WWTP with an extremely low abundance (0.01% average). The identified anammox bacteria belonged to the genus of *Candidatus Brocadia* in the phylum *Planctomycetota*, which contained the first discovered anammox organism (*Candidatus Brocadia anammoxidans*).

#### AOB is more diverse than NOB for both Hong Kong and global WWTPs

To have a deeper understanding of the nitrification process of Hong Kong and worldwide WWTPs, we examined AOB and NOB conditions in a worldwide survey for 269 WWTPs with 1,186 AS samples and then compared their results with Hong Kong WWTPs (Fig. S6; Table S6). In total, 358 AOB OTUs were defined for worldwide WWTPs with ~67% of these AOB OTUs did not have the genome information yet (Table S7). These 358 AOB OTUs are distributed in *f_Nitrosomonadaceae*, *g_Nitrosococcus*, *g_Nitrosospira,* and *g_Nitrosomonas*. Besides, Hong Kong WWTPs had four unique AOB OTUs which were not detected in worldwide WWTPs (Fig. S6), although they all had low relative abundance. More interestingly, these four AOB OTUs were only detected in SWH WWTPs, indicating the special ecological niche of the AOB community in SWH WWTPs for nitrification. Moreover, it is challenging to identify a dominant AOB across WWTPs since the type and abundance of AOB exhibit a considerable amount of diversity among different WWTPs as revealed by the PCoA analysis in Fig. S7. This scenario is consistent with the results we observed in Hong Kong WWTPs.

In all, 310 NOB OTUs were detected in worldwide WWTPs, including *c_P9 × 2b3D02*, *g_Nitrococcus*, *g_Nitrolancea*, *g_Candidatus Nitrotoga,* and *g_Nitrospira*. The *g_Nitrospira* was identified to be the most abundant NOB in not only this study but also another research of global WWTPs (66). Among these 310 NOB OTUs, 65% of them did not have genome information yet (Table S7), indicating the importance of obtaining complete/high-quality genomes of these key species in WWTPs. Unlike AOB, NOB shows a high degree of consistency across different WWTPs with the consequence that a *Nitrospira* OTU (OTU_6) was identified as the core taxon for worldwide WWTPs (16) by showing in 1,057 out of 1,186 WWTPs with the average and highest relative abundance of 0.9% and 7.7%, respectively. It was also the dominant NOB for Hong Kong WWTPs (*Nitrospira_2*) with resolved genome information.

AOB appears to exhibit greater diversity and discrepancy than NOB in both Hong Kong and worldwide WWTPs: the types and quantities of AOB in different WWTPs vary greatly. Wastewater has relatively complex sources and violently fluctuating water quality with dynamic physiochemical parameters. AOB is the first executor of nitrification and high diversity could facilitate its rapid adaption to various conditions of wastewater by exploring different niches in the AS community to facilitate the nitrification process. The role of NOB is to oxidize nitrite produced by AOB, which means NOB can experience a more stable reaction environment after the performance of the AOB “buffer.” Therefore, the mainstream NOB strains are relatively uniform across different WWTPs. The condition that the AOB community was more diverse, resistant, and resilient than the NOB community was also reported in both WWTPs (67, 68) and nitrifying bioreactors (69, 70) by other researchers when they assessed the dynamic of the microbial population. In fact, the situation that the diversity of bacteria performing the first step is higher than that of the second/following step is common in the multi-step microbial reaction. For example, the methanogenic process contains four steps: hydrolysis, fermentation, syntrophic oxidation, and methanogenesis (71). Basically, the species and abundance of bacteria with the potential to proceed with each process gradually decreased and only a limited microbial group could complete the final methanogenesis step (72, 73).

#### Abundance and distribution of PAO groups in Hong Kong and global WWTPs

Another important function of WWTPs is the removal of phosphorus, which is mainly completed by PAOs, a group of bacteria that can facilitate the removal of large amounts of phosphorus from wastewater by accumulating phosphorus within their cells as polyphosphate (19). *Candidatus Accumulibacter* (*Ca. Accumulibacter*) (74) and *Tetrasphaera* (75) are two kinds of PAOs whose phosphorus removal abilities have been identified by researchers. In summary, five *Ca. Accumulibacter* and two *Tetrasphaera* OTUs presented in Hong Kong WWTPs and the *Ca. Accumulibacter* had a higher abundance than *Tetrasphaera* (Fig. S8). Among them, *Ca. Accumulibacter_1* was the key PAO in Hong Kong WWTPs that showed in three WWTPs and had the highest average abundance of 1.03% in SWH WWTP. With the wider distribution, *Tetrasphaera_1* existed in all WWTPs and showed the highest average abundance of 0.31% in ST WWTP. In all, 92 *Ca. Accumulibacter* and 33 *Tetrasphaera* were found in worldwide WWTPs with the fact that *Ca. Accumulibacter* had more diversity while *Tetrasphaera* was more prevalent (Table S8). According to the results of global data and other studies, PAOs could have >5.02% abundance in many countries that apply the enhanced biological phosphorus removal (EBPR) technology, such as Japan, Denmark (16), and Italy (75). Compared to the results of worldwide WWTPs, the abundance of PAOs was not high in Hong Kong mainly due to the lack of phosphorus removal process (i.e., EBPR) in WWTP design.

#### Abundance and distribution of BFB groups in Hong Kong and global WWTPs

BFB groups are always present in AS and might harm wastewater treatment systems (76). Seven kinds of BFB were detected in six Hong Kong WWTPs, including *Acinetobacter* sp., *Caldilinea* sp., *Gordonia* sp., *Moraxella osloensis*, *Mycobacterium* sp., *Skermania piniformis,* and *Thiothrix* sp. (Fig. S9). The most universal BFBs in Hong Kong WWTPs were *Acinetobacter* sp. and *Mycobacterium* sp., and these two BFBs were persistent in AS and detected in all WWTPs. The highest average abundance of *Acinetobacter sp*. and *Mycobacterium* sp. both showed in STL WWTP with an abundance of 1.22% and 0.90%, respectively. Among six Hong Kong WWTPs, STL WWTP suffered the most serious BFB problem with the highest type (five types) and abundance (0.34% average) of BFB, while SWH and YL WWTPs were much lower influenced by BFB compared with other WWTPs. Another important and common BFB that is worthy of attention is *Gordonia* sp. We detected *Gordonia* sp. in four Hong Kong WWTPs (SK, ST, STL, and TP) and further results showed *Gordonia* sp. seemed to have seasonal dynamic changes, that is, it had higher abundance in winter-spring while lower abundance in summer-autumn. This situation was also observed in a 5-year BFB survey of ST WWTP (77).

In total, 29 genera BFB (Table S9) were detected in global WWTPs with the top three genera of *Acinetobacter* (170 OTUs), *Defluviicoccus* (72 OTUs), and *Thiothrix* (63 OTUs). *Acinetobacter* was the most diverse BFB for both Hong Kong and global WWTPs, which showed in almost all WWTPs (99.3%) and had an average abundance of 0.22%. In addition to being a notorious foaming bacterium, *Acinetobacter* is a potential environmental pollutant since it contains antibiotic resistance genes and can be pathogens (78). Compared to filamentous bacteria, it is more common to study *Defluviicoccus* as the glycogen accumulating organisms, which shows in not only EBPR communities but also WWTPs operated at high temperatures or with high industrial loads (79). *Thiothrix* is also a common BFB and is detected in global WWTPs with an abundance of up to 41.3% in Santiago, Chile. Actually, the abundance of some BFB genera could be underestimated due to V4 primer bias (66), such as *Leptothrix* and *Sphaerotilus*. Interestingly, researchers reported that the growth of some BFB was related to particular treatment processes of wastewater. For example, *Ca. Microthrix* obtains good growth under microaerophilic conditions and rarely shows in WWTPs that are designed to remove carbon only (80, 81).

In fact, all the above discussions are based on the relative abundance of the 16S rRNA gene. It is more meaningful to count functional bacteria at the cell level with absolute quantification, especially for an engineering system like AS. The 16S rRNA gene has multiple copies in cells and amplification bias generated by PCR may mislead our judgment of ecological questions by providing distorted quantitative relationships. Although many tools like rrnDB (82) are available to conduct the correction, we have to be careful about the resulting false positives when trying to convert 16S rRNA gene numbers to cell numbers (83). Absolute quantification has critical significance in microbial research when researchers want to compare samples collected from different sites like this study or long-term studies. The enrichment of taxa (increase in relative abundance) does not necessarily relate to the outgrowth of taxa (increase in absolute abundance) (84), and thus direct comparisons of relative abundance for different samples may lead to wrong conclusions.

### The MDM conditions for Hong Kong and global WWTPs

**Fig 5:**
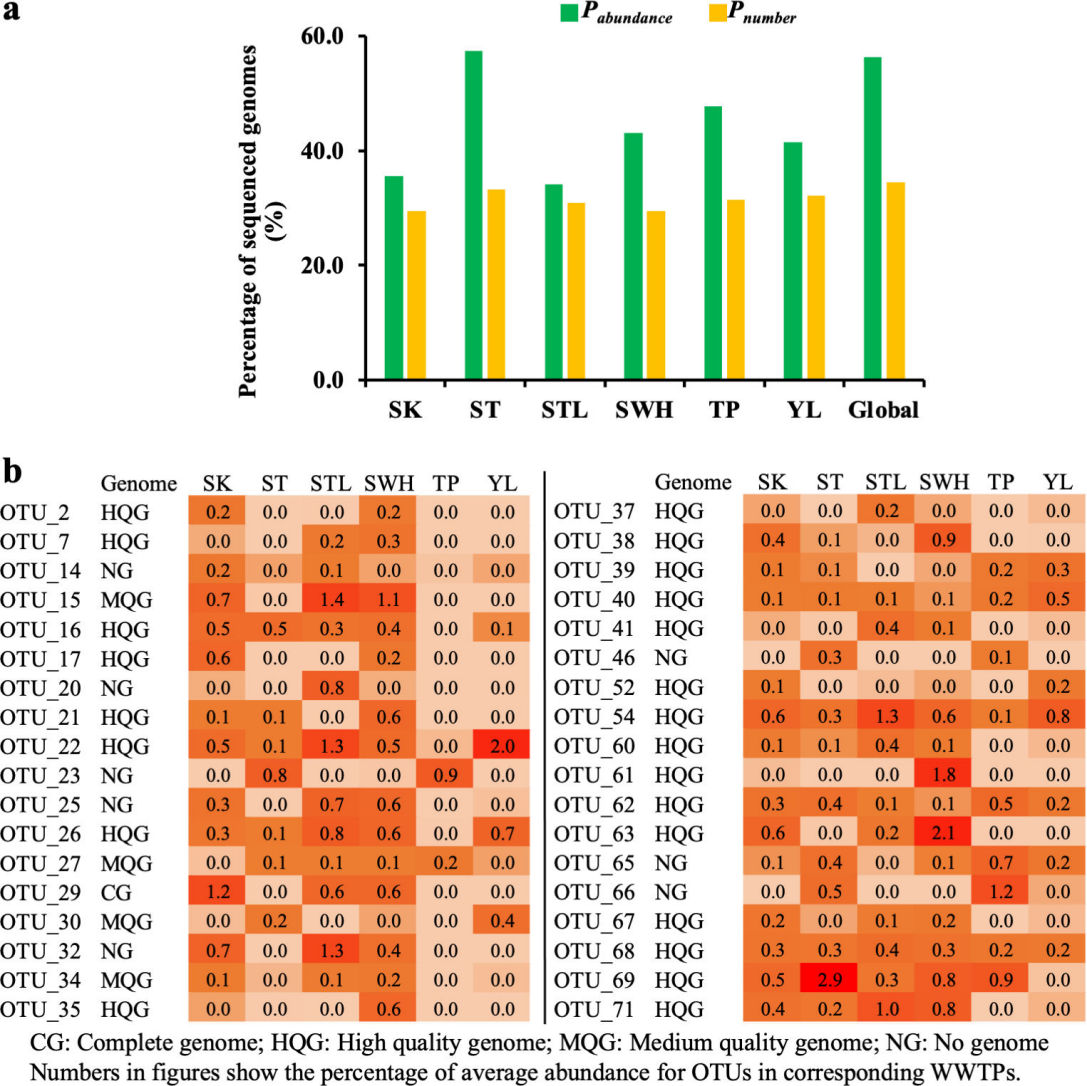
MDM conditions of six Hong Kong WWTPs. (**a**) *P*_number_ and *P*_abundance_ results of Hong Kong and global WWTPs. *P*_number_ and *P*_abundance_ were used to represent the genome-sequenced proportions of taxa (total OTU number) number and corresponding cell number (total relative abundance sum) sum. (**b**) Distribution of 36 OTUs from global “wanted list” in Hong Kong WWTPs.

Our previous research defined the “wanted list” of global WWTPs and revealed that the median proportions of the genome-sequenced cells and taxa (100% identity and 100% coverage in 16S rRNA gene region) in global AS reached 56.3% and 34.5%, respectively (23). Fig. 5a shows ST WWTP had the highest genome-sequenced proportions among six Hong Kong WWTPs since the deep sequencing (up to 500 Gb) on ST AS samples had been performed to obtain numerous high-quality and complete genomes by the hybrid assembly of short/long reads (85). The genome-sequenced proportions of TP and YL WWTPs were also high since they had similar bacterial flora structures with ST WWTP. The remaining three WWTPs of SK, STL, and SWH had lower genome-sequenced proportions than global WWTPs (23), indicating the fact that the great majority of species in Hong Kong WWTPs lack physiological characteristics and only a few microorganisms could be characterized based on available genomes and pure cultures. For the distribution of the global “wanted list” in Hong Kong WWTPs, 36 out of 71 OTUs that do not have isolates yet could be found with >0.1% average relative abundance (Fig. 5b; Table S10). For instance, OTU_69 and OTU_63 had an average relative abundance of 2.9% in ST WWTP and 2.1% in SWH WWTP, respectively. With such a high abundance, Hong Kong WWTPs could make great contributions to the genome mining of microbiota in AS by both sequencing and isolation (86), especially for the eight OTUs that do not have any genome information in the present database.

Although, already, there have been lots of microbiome surveys in WWTPs, the importance of sub-genus level diversity in the AS ecosystem is largely unknown. Since functional traits are often conserved at low taxonomic ranks (87), knowledge about the role of individual species in the ecosystem is of crucial importance to understanding the ecosystem function, resilience to disturbances, and biological nutrient transformation routes. The significance of species-level microbiome composition was emphasized by recent studies. For instance, *comammox* (88) and *Tetrasphaera* (89) contain certain crucial important functions to the ecosystem, and these functions are only exhibited by some members of the genus. Partial regions of the 16S rRNA gene provide poor resolution at the species level, while scientists have taken note of this and have built many databases such as GTDB (35), the ribosomal RNA operons (90), and the full-length 16S rRNA gene (91).

### The effects between operational parameters and OTUs’ abundance for Hong Kong and global WWTPs

**Fig 6:**
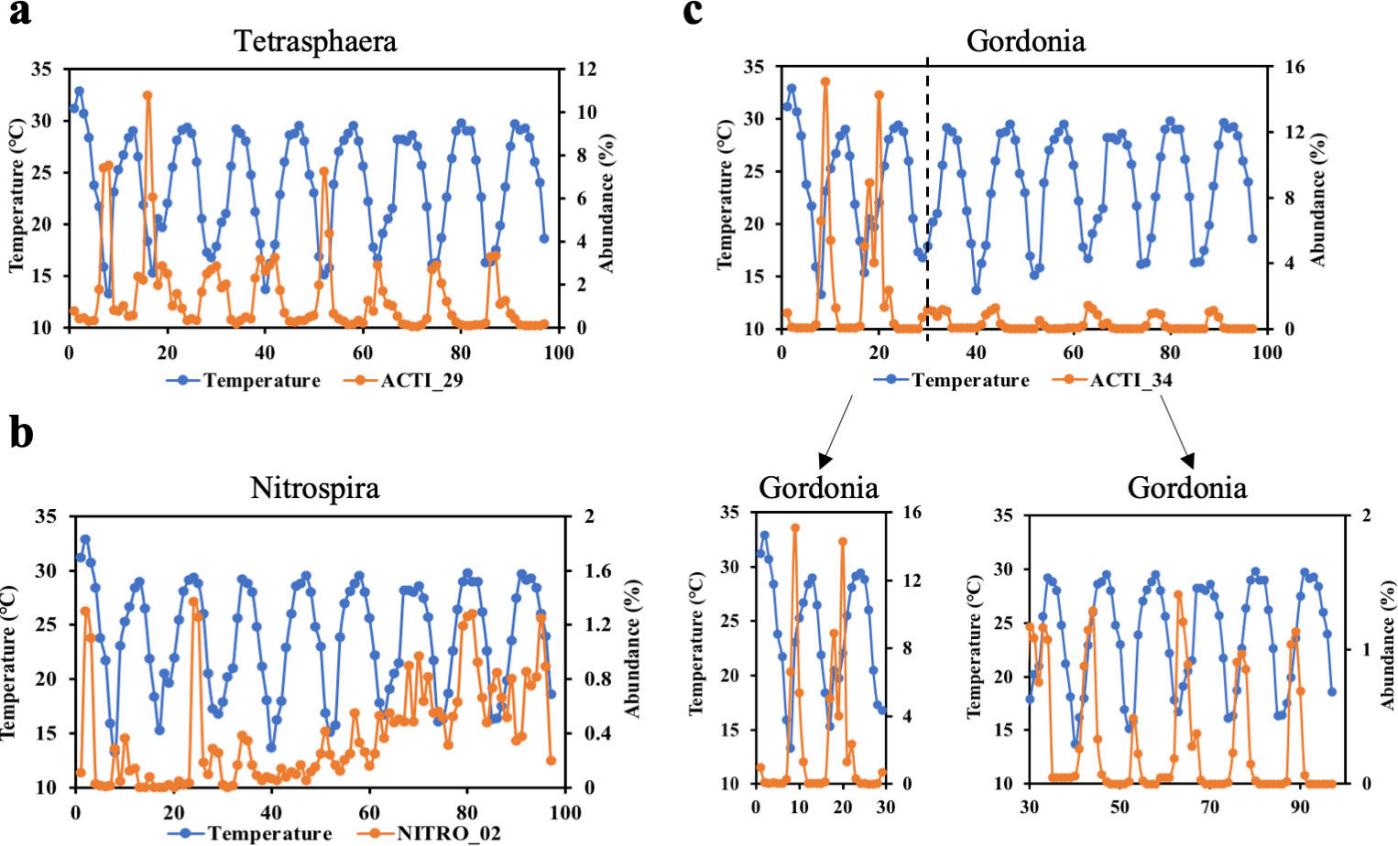
Relationships of temperature and abundance of MAGs in ST AS with over 9-year data. Three MAGs were included: (a) *Tetrasphaera*, (b) *Nitrospira,* and (c) *Gordonia*.

The RDA was conducted between functional OTUs and operational parameters for Hong Kong WWTPs to highlight the top three important operational parameters: temperature, mean cell residence time, and hydraulic retention time (Fig. S10). Figure S10 shows the temperature could affect the abundance of *Tetrasphaera*, *Nitrospira,* and *Gordonia* in six Hong Kong WWTPs. To further verify the relationships between bacteria and operational parameters with long-term data, we extracted the metagenomic sequencing data of ST AS (over 9 years) from the former research (40) to show the correlation of temperature and abundance with three MAGs (Fig. 6; Table S11). The situation that *Tetrasphaera* (Fig. 6a) has a negative relationship with temperature has been widely observed and reported in global WWTPs (92, 93). It seems that a temperate environment (10°C–20°C) probably supports the proliferation of *Tetrasphaera*, but *Tetrasphaera* may fail the growing competition with other microbes under high temperatures (75). According to Fig. 6b, the abundance of *Nitrospira* had a positive relationship with temperature, which indicated *Nitrospira* could have a better performance under high temperatures and provided a possible explanation for nitrification failure in winter with solid evidence (24). Different from *Nitrospira*, *Gordonia* (Fig. 6c) had a high abundance under low temperatures, which was consistent with our observation that sludge foaming had a high frequency in winter for Hong Kong (1) and other WWTPs (94).

Actually, the abundance change in the microbial community is the interaction consequence of many operational parameters and it is particularly difficult to elaborate on the influence of one operational parameter specifically. More long-term data on global WWTPs are needed to track continuous dynamic changes of specific bacteria in one WWTP and verify stable relationships in multiple WWTPs with different treatment processes and operational parameters. RDA in this study could provide a solution to find potential crucial parameters for technical improvements and more data analysis tools should be applied to reveal key connections among the community in the big data era. For instance, researchers tried to predict the community composition with machine/deep learning models trained by environmental parameters (27, 95, 96). All this valuable information can help engineers understand microbial relationships deeply and control the performance of WWTPs better.

### Conclusions

A 13-month bacterial community survey of six full-scale WWTPs in Hong Kong was conducted and results were compared to data from worldwide WWTPs and the long-term survey of ST WWTP to get the following conclusions.

- The AS bacterial community structures of six Hong Kong WWTPs were markedly distinct from one another.
- Forty pairs of co-occurrence relationships identified in multiple WWTPs revealed widespread microbial associations in AS.
- Stochastic processes took large proportions for the bacterial community assembly.
- The abundance and distribution of the functional bacteria in worldwide and Hong Kong WWTPs were different. The AOB was more diverse than NOB for both Hong Kong and worldwide WWTPs. This fact may reveal a fundamental truth that the functional bacteria responsible for downstream reactions in multi-step biochemical processes are more conserved.
- Of 71 OTUs in the global “wanted list,” 36 did not have isolates yet but had >0.1% average abundance in Hong Kong WWTPs.
- Operational parameters had important effects on OTUs’ abundance for both Hong Kong and worldwide WWTPs, such as the temperature to the genera of *Tetrasphaera*, *Gordonia* and *Nitrospira*.
- Further research could reveal novel insights into bacterial communities in WWTPs with tools of community assembly pipelines at the lineage level, absolute qualification and machine learning.

## Data Availability

The raw data of 16S rRNA reads could been downloaded from NCBI with the BioProject ID PRJNA1012295.

## SUPPLEMENTAL MATERIAL

Supplemental material msystems.01333-23-s0001.pdf Supplemental text and Figures S1-S13.

Supplemental tables msystems.01333-23-s0002.xlsx Tables S1-S11.

ASM does not own the copyrights to Supplemental Material that may be linked to, or accessed through, an article. The authors have granted ASM a non-exclusive, world-wide license to publish the Supplemental Material files. Please contact the corresponding author directly for reuse.
