# Supplementary material for: Effects of operational parameters on bacterial communities in Hong Kong and global wastewater treatment plants
Source: mSystems. 2024 Feb 27;9(3):e01333-23. doi: 10.1128/msystems.01333-23 (PMC10949511; doi:10.1128/msystems.01333-23)
Supplement: Supplemental material — Supplemental text and Figures S1-S13. [file msystems.01333-23-s0001.pdf]

Effects of Operational Parameters on Bacterial Communities in Hong Kong and Global Wastewater Treatment Plants

Yulin Zhang [a†], Yu Deng [a†], Chunxiao Wang [a], Shuxian Li [a], Frankie T.K. Lau [b], Jizhong Zhou [c], Tong Zhang [a, d*]

[a] Environmental Microbiome Engineering and Biotechnology Lab, Department of Civil Engineering, The University of Hong Kong, Pokfulam Road, Hong Kong, China

[b] Drainage Services Department, The Government of the Hong Kong Special Administrative Region of the People's Republic of China, Wanchai, Hong Kong, China

[c] Institute for Environmental Genomics, Department of Microbiology and Plant Biology, and School of Civil Engineering and Environmental Sciences, University of Oklahoma, Norman, OK, USA

[d] Macau Institute for Applied Research in Medicine and Health, Macau University of Science and Technology, Macau, China

[†] Co-first author

* Corresponding author. Phone: +852-28578551. Fax: +852-25595337. E-mail: zhangt@hku.hk.

This supporting material has 19 pages, including 13 figures.

The variation of alpha diversity for 6 Hong Kong WWTPs

The performance of WWTPs could be more stable if AS communities have higher diversity [1], which is usually indicated by alpha diversity. Alpha diversity refers to the richness (the number of unique detected bacteria, Chao 1), diversity (abundance of each species, Shannon and Simpson) and evenness (similar abundance level, Pielou's evenness) of bacterial community within a single sample. As shown in Figure 1a, Chao 1 index ranged from 541 (201801_YL_WWTP) to 1665 (201809_SWH_WWTP) in all samples. The Shannon index varied from 4.70 (201808_YL_WWTP) to 8.73 (201811_SWH_WWTP) in these time series samples. Besides, Simpson diversity index ranged from 0.81 (201808_YL_WWTP) to 0.99 (201811_SWH_WWTP) and Pielou's evenness index varied from 0.51 (201808_YL_WWTP) to 0.84 (201811_SWH_WWTP). Among the 6 WWTPs, AS communities from SWH WWTP had the highest richness and diversity while YL WWTP had the lowest ones. Besides, previous studies indicated the importance of temperature for the shape of community diversity in different seasons and the seasonal succession of bacterial communities over various environmental gradients has been widely observed in many ecosystems, including soil [2], lakes [3] and WWTPs [4]. Here, we compared the seasonal variations in bacterial diversity between time-series AS samples with Kruskal-Wallis H test for the four diversity indices of each WWTP. Results showed that there was no significant difference in community richness for different seasons, partly due to the subtropical climate of Hong Kong that had limited temperature fluctuation throughout the year. But operational parameters may have impacts on the diversity of communities (Figure S11) through the redundancy analysis.

The potential influence of operational parameters on the diversity of bacterial community for HK WWTPs

Although the microbial diversity of AS is relatively stable in WWTPs, it still has little fluctuation that might be influenced by multiple factors like environmental conditions, water quality, and operational parameters. Redundancy analysis (RDA) was conducted to determine potential operational parameters that may influence communities. The operational parameters include weather information like temperature and humidity, sludge physicochemical information such as mean cell residence time (MCRT) and sludge volume index (SVI), and sewage information like NO_3-N. Results (Figure S11) showed that different alpha-diversity indexes had distinct positive or negative correlations to operational parameters in different WWTPs. For instance, the impact degree ranking of the four alpha-diversity indexes of SK WWTP is Shannon > Simpson > Pielou's evenness > Chao1. Overall, Shannon index was most likely influenced by operational parameters while Chao1 index had the least interference. Besides, statistical analysis indicated that some operational parameters (MCRT, OP.P, and NO2.N) could influence (P<0.05) bacterial diversity of ST WWTP. Collectively, these results suggested that the microbial diversity of 6 WWTPs had relatively stable diversity patterns throughout the year with no obvious seasonal difference but could be impacted by operation parameters.

The dynamics of the community structure of AS for HK WWTPs

As an artificially controlled engineered biological system, the dynamics of the community structure of AS for Hong Kong WWTPs were evaluated. Figure 1b indicated there was no obvious temporal succession of microbial community composition for AS in Hong Kong except for ST WWTP, showing the stability of community structure in AS for the five WWTPs (SK, STL, SWH,

TP, YL). The stable community structures of five WWTPs were consistent with the conclusion made by other researchers that AS community had a relatively stable pattern throughout the year [4]. Besides, the dynamic change of community structure for ST WWTP indicated the potential community shift. A similar result of ST WWTP was also observed by a previous study, which conducted a 5-year survey for AS of ST WWTP and found the temporal changes in the phylogenetic composition and abundance of OTUs were quite high [5]. This situation might be induced by fluctuations of chemical substances in influent as ST WWTP is the largest secondary WWTP in Hong Kong and the specific operating parameters.

The core community of 6 WWTPs

We defined the core community with three indexes: universality, abundant, and dominant. To get more concise lists, we further narrowed the cutoff of core species in the main text. The core OTUs should be detected in more than 80% of total samples with a relative abundance >0.5% for more than 50% of total samples. Figure S12a indicated that STL WWTP had the largest number of core species with 41 OTUs while ST WWTP had the lowest number of 29 OTUs. What's more, few core species were shared among different WWTPs, except for the 17 OTUs overlap between ST and TP, and 16 OTUs shared for SWH and SK. This situation is highly consistent with the analysis results of community compositions of Hong Kong WWTPs and also matches the conclusions of global WWTPs. Previous studies have reported the core community in WWTPs at regional scales, such as core genera that exist in Danish [6], Asian [7] and global WWTPs [8], and found core communities of different regions had less overlap. We then analyzed the occupancy-frequency and occupancy-abundance analyses of the core community and revealed a hyper-dominant pattern (Figure S12b) of all 6 WWTPs in which the small number of the core community accounted for

large proportions of the total abundance. For example, the core community of ST WWTP occupied 3.3% of the total OTUs number while they accounted for 45.7% of the total abundance. Similar hyper-dominance patterns were also observed in the global WWTPs [8] and other microbiological communities like soil.

In general, these core species play important roles during wastewater treatment. Most of the core species in HK WWTPs belong to the phyla of *Proteobacteria* and *Bacteroidete*, two of the common phyla in WWTPs. Besides, core OTUs belonging to the genus of *Nitrosomonas* and *Nitrospira* showed in core lists to conduct nitrogen removal in WWTPs. Other functional bacteria like *Candidatus Accumulibacter* were also identified in core groups. Considered to be a highly functional redundant system [9], AS system contains different microorganisms that may play the same ecological roles, which may explain the unique core community detected in different WWTPs. We also examined the relationships between core communities and operational parameters and found that the abundance of core communities could be affected by operational parameters like temperature (Figure S13).

Reference

1. Zhang, B., et al., *Biodegradability of wastewater determines microbial assembly mechanisms in full-scale wastewater treatment plants.* Water Research, 2020. **169**: p. 115276.
2. Lipson, D.A. and S.K. Schmidt, *Seasonal changes in an alpine soil bacterial community in the colorado rocky mountains.* Appl Environ Microbiol, 2004. **70**(5): p. 2867-79.
3. Eiler, A., F. Heinrich, and S. Bertilsson, *Coherent dynamics and association networks among lake bacterioplankton taxa.* The ISME Journal, 2012. **6**(2): p. 330-342.
4. Sun, C., et al., *Seasonal dynamics of the microbial community in two full-scale wastewater treatment plants: Diversity, composition, phylogenetic group based assembly and co-occurrence pattern.* Water Research, 2021. **200**: p. 117295.
5. Ju, F. and T. Zhang, *Bacterial assembly and temporal dynamics in activated sludge of a full-scale municipal wastewater treatment plant.* The Isme Journal, 2014. **9**: p. 683.
6. Saunders, A.M., et al., *The activated sludge ecosystem contains a core community of abundant organisms.* The ISME Journal, 2016. **10**(1): p. 11-20.
7. Zhang, T., M.-F. Shao, and L. Ye, *454 Pyrosequencing reveals bacterial diversity of activated sludge from 14 sewage treatment plants.* The ISME Journal, 2011. **6**: p. 1137.
8. Wu, L., et al., *Global diversity and biogeography of bacterial communities in wastewater treatment plants.* Nature Microbiology, 2019. **4**(7): p. 1183-1195.
9. Vuono, D.C., et al., *Disturbance and temporal partitioning of the activated sludge metacommunity.* The ISME Journal, 2015. **9**(2): p. 425-435.

Figure

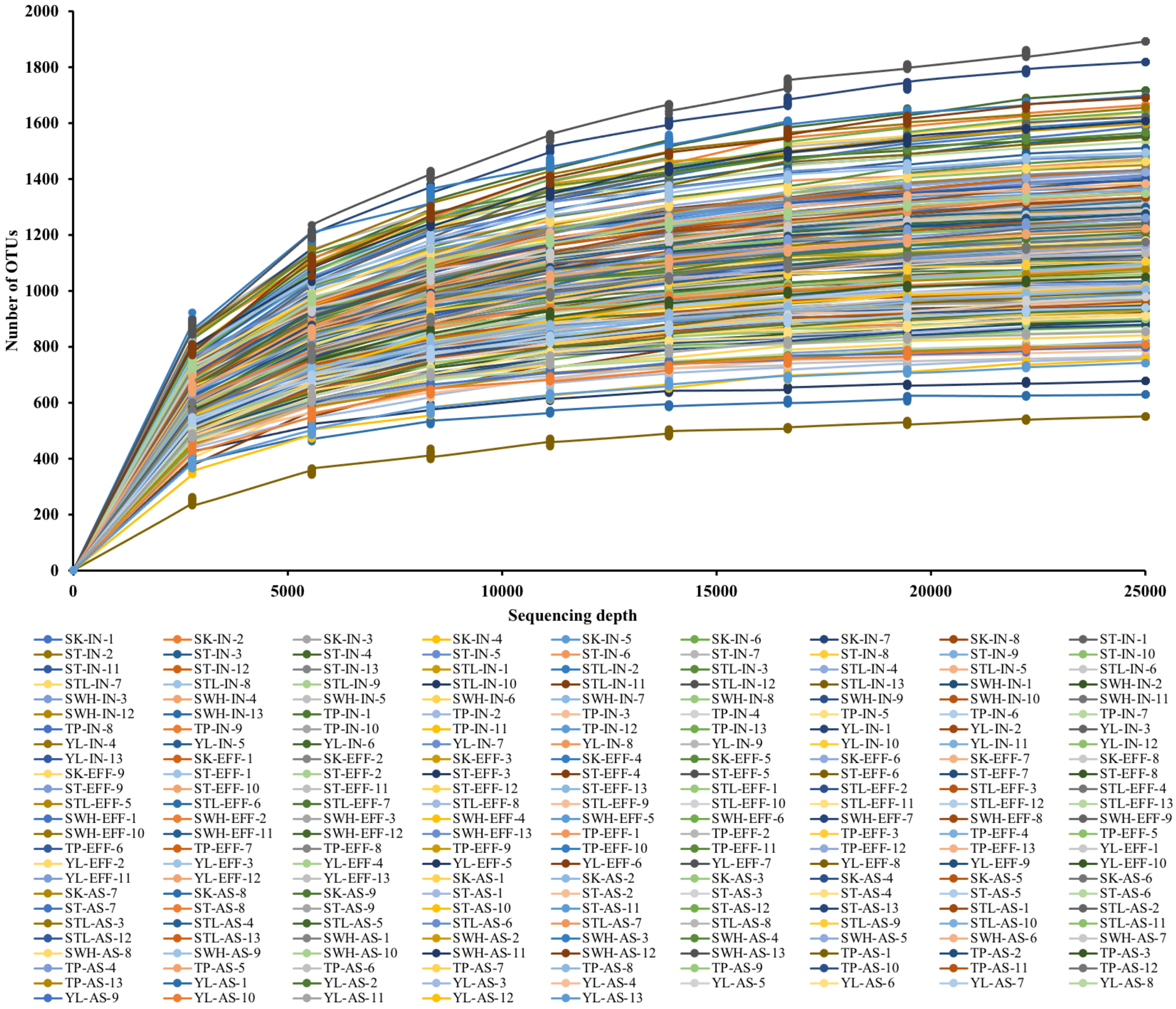

Figure S1 Rarefaction curve of samples for 6 WWTPs. IN: influent; AS: activated sludge; EFF: effluent.

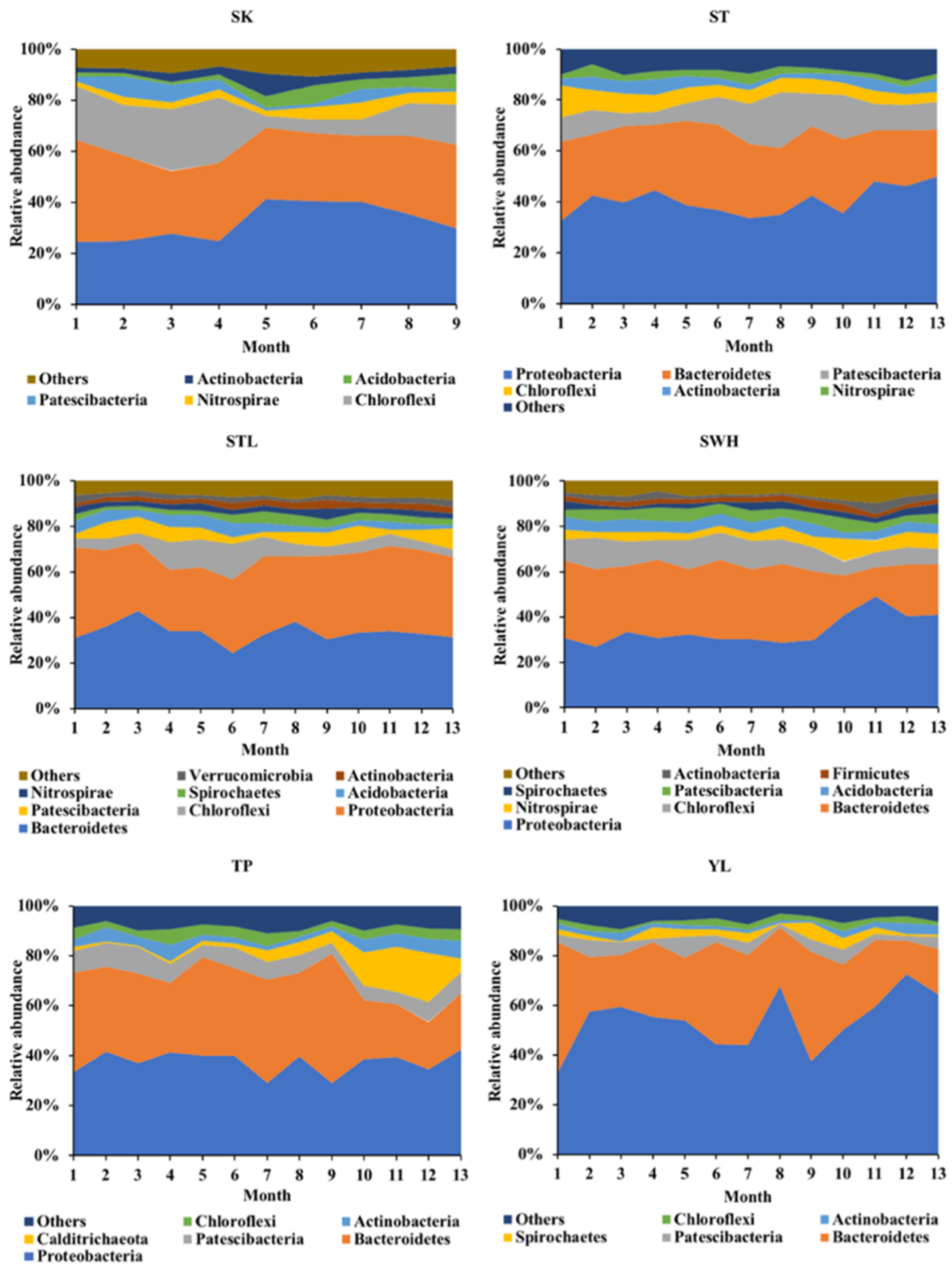

Figure S2 The taxonomic composition of microbial community for 6 WWTPs. The figure only showed phyla whose relative abundance was >2%.

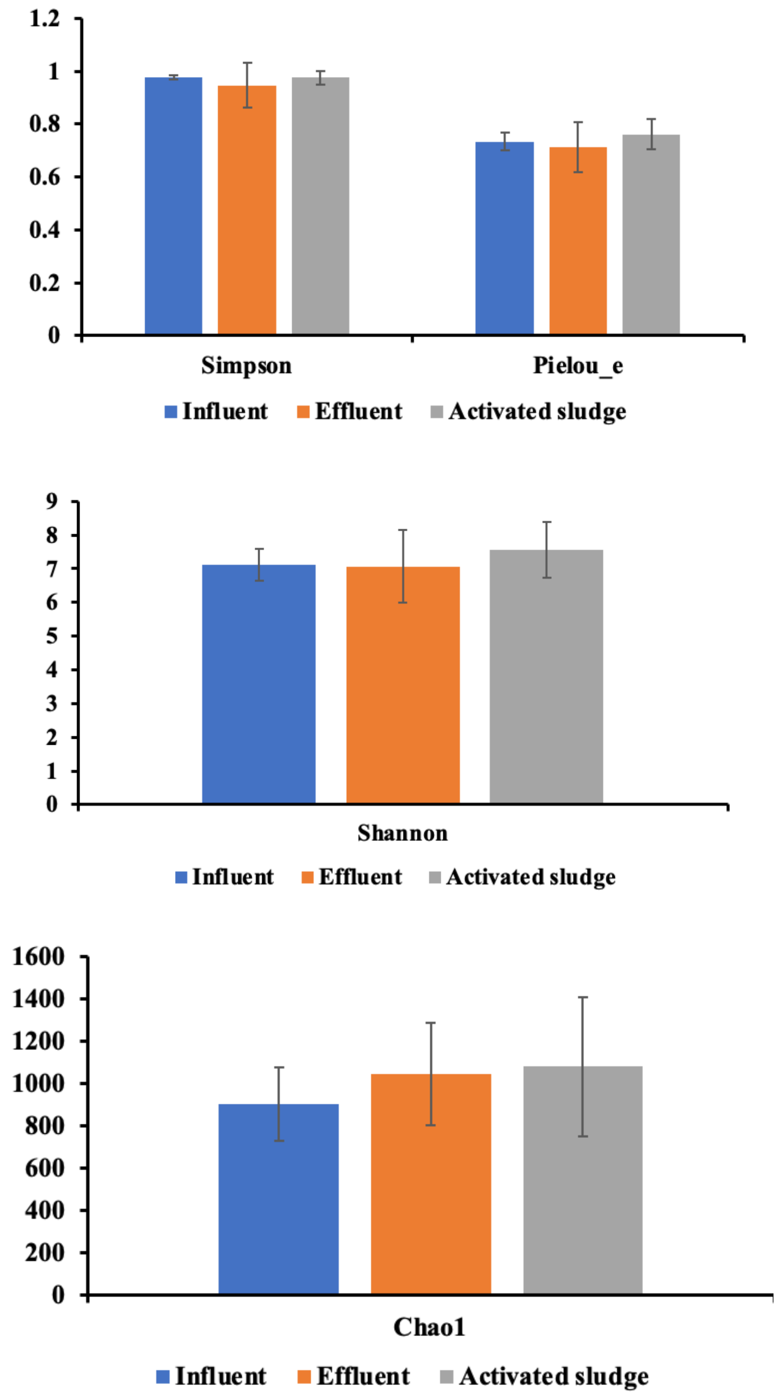

Figure S3 The average value and standard deviation of α diversity for influent, activated sludge and effluent samples.

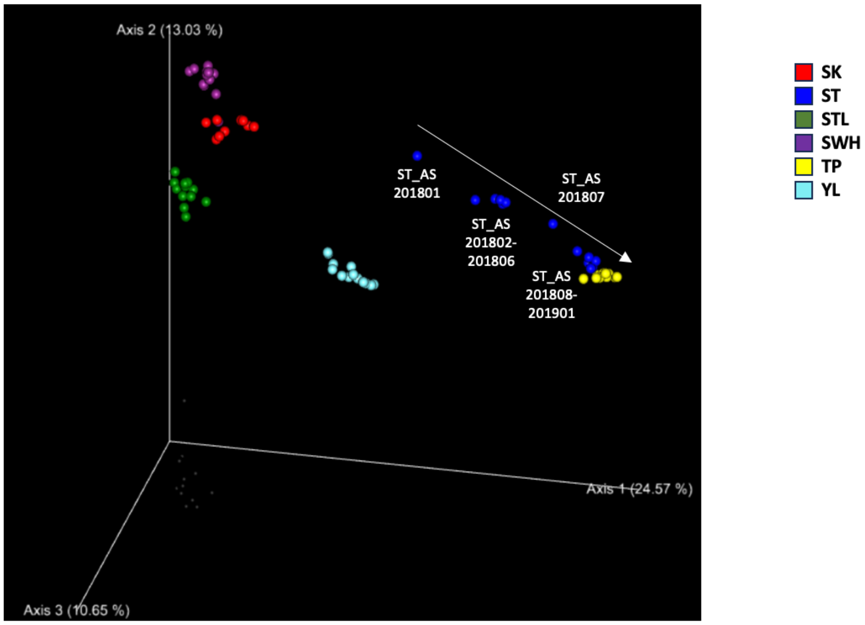

Figure S4 The PCoA plot of AS conducted by comparing pairwise microbial community dissimilarity distance using Bray-Curtis algorithm. Each dot represented the microbial community structure of an individual samples. Colors represented different WWTPs.

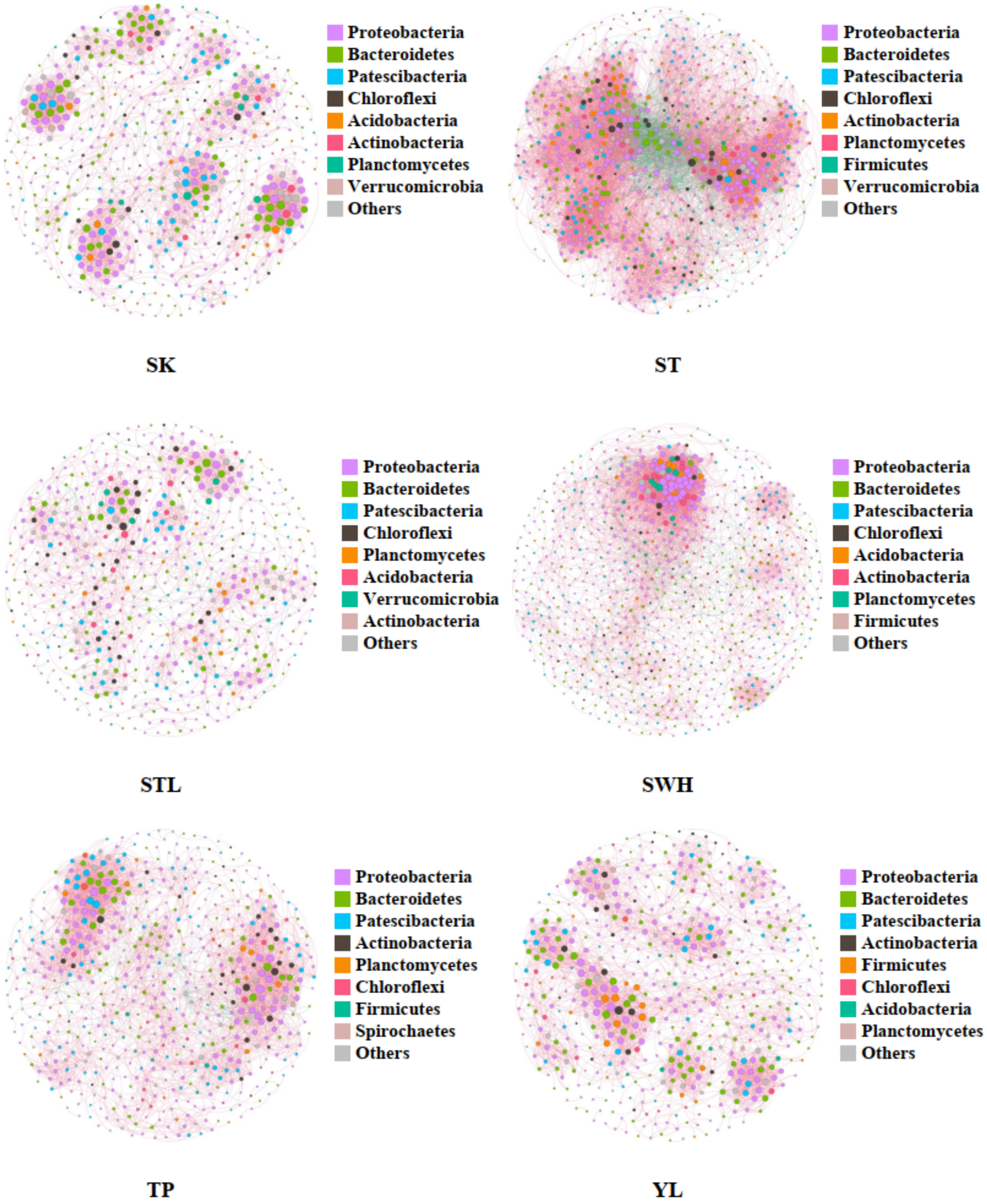

Figure S5 Network patterns of 6 WWTPs based on pair-wise Spearman's rank coefficients. Red and green colors represented positive and negative relationships, respectively. Each node represented each OTU. Their sizes were determined by the frequency of OTUs and their colors were used to annotate the taxonomy information in different WWTPs.

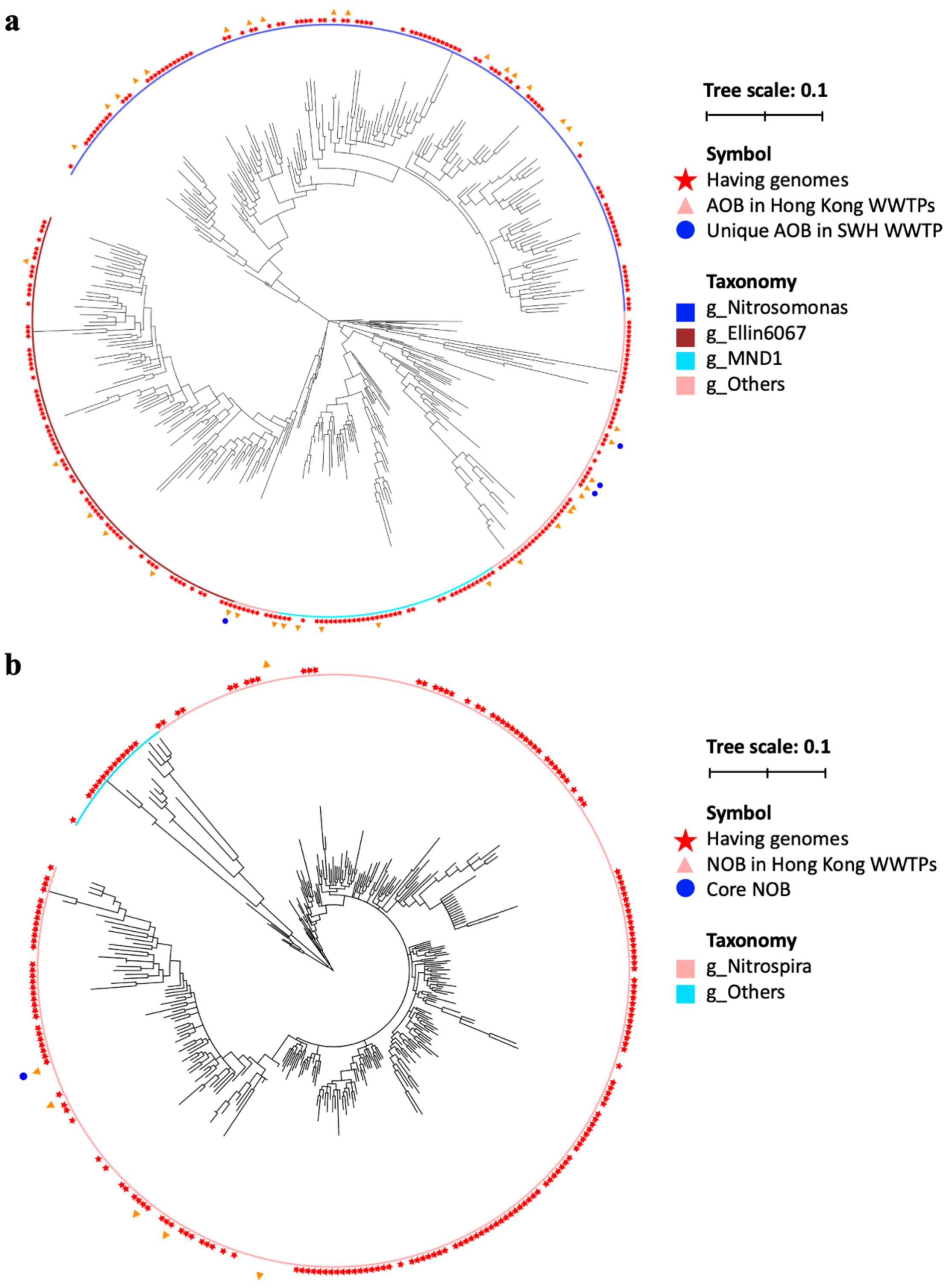

Figure S6 The phylogenetic tree of a) AOB and b) NOB for Hong Kong and global WWTPs.

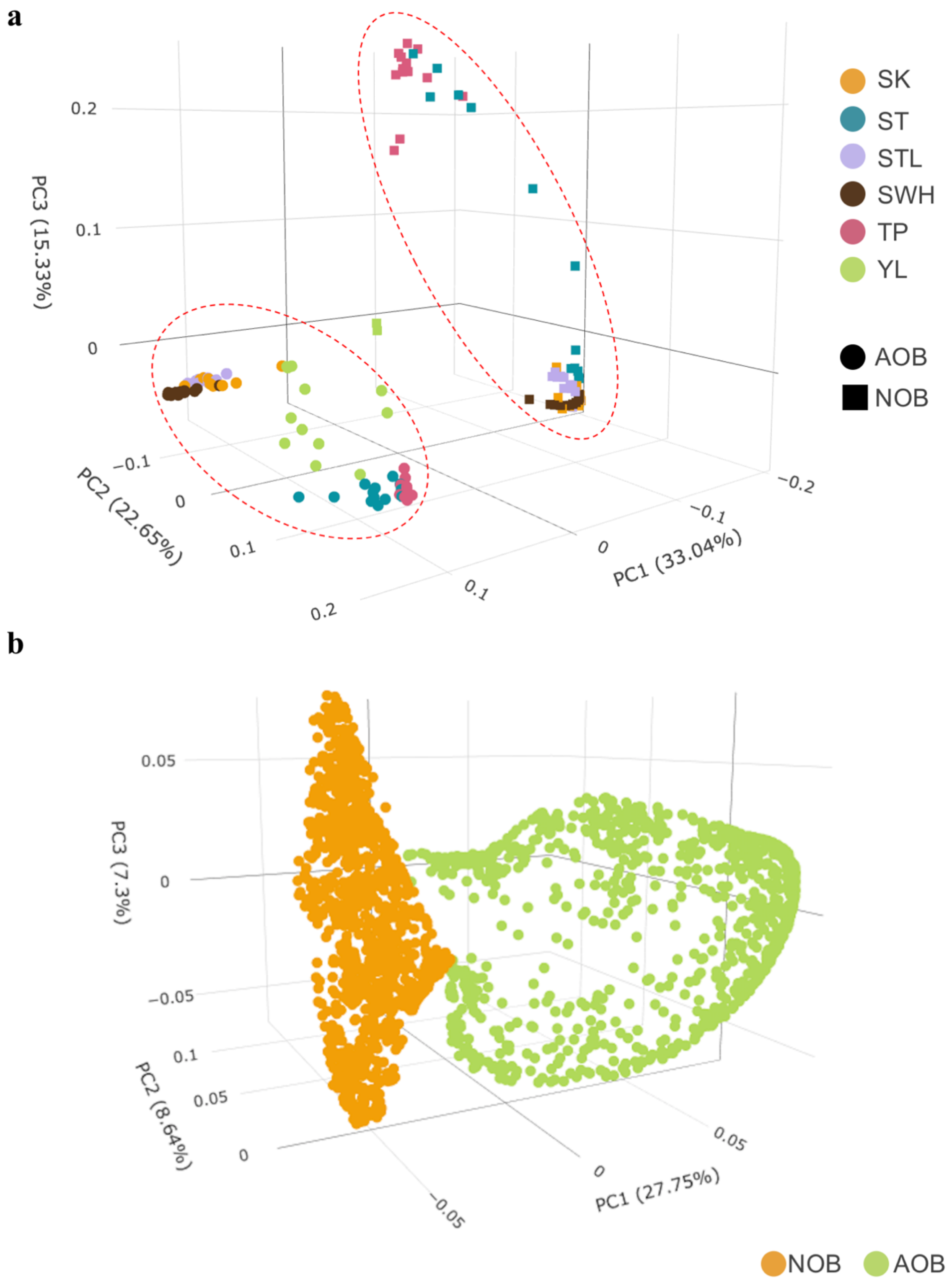

Figure S7 The PCoA plot of AOB and NOB for a) Hong Kong and b) worldwide WWTPs.

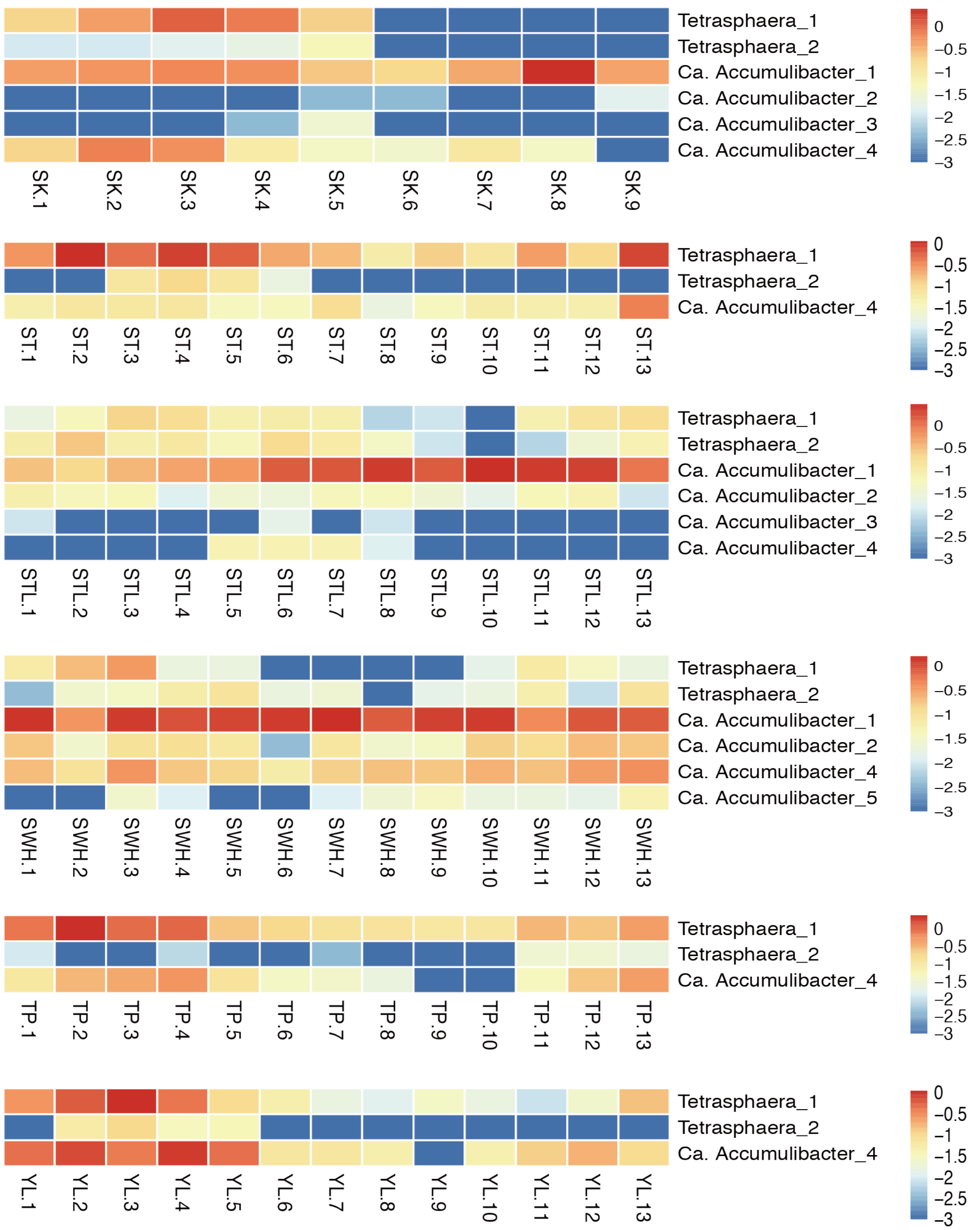

Figure S8 Compositions, abundance, and distributions of PAOs in 6 WWTPs of Hong Kong. The abundance data were treated by log_{10}. Only the OTUs with the average abundance >0.01% were reserved.

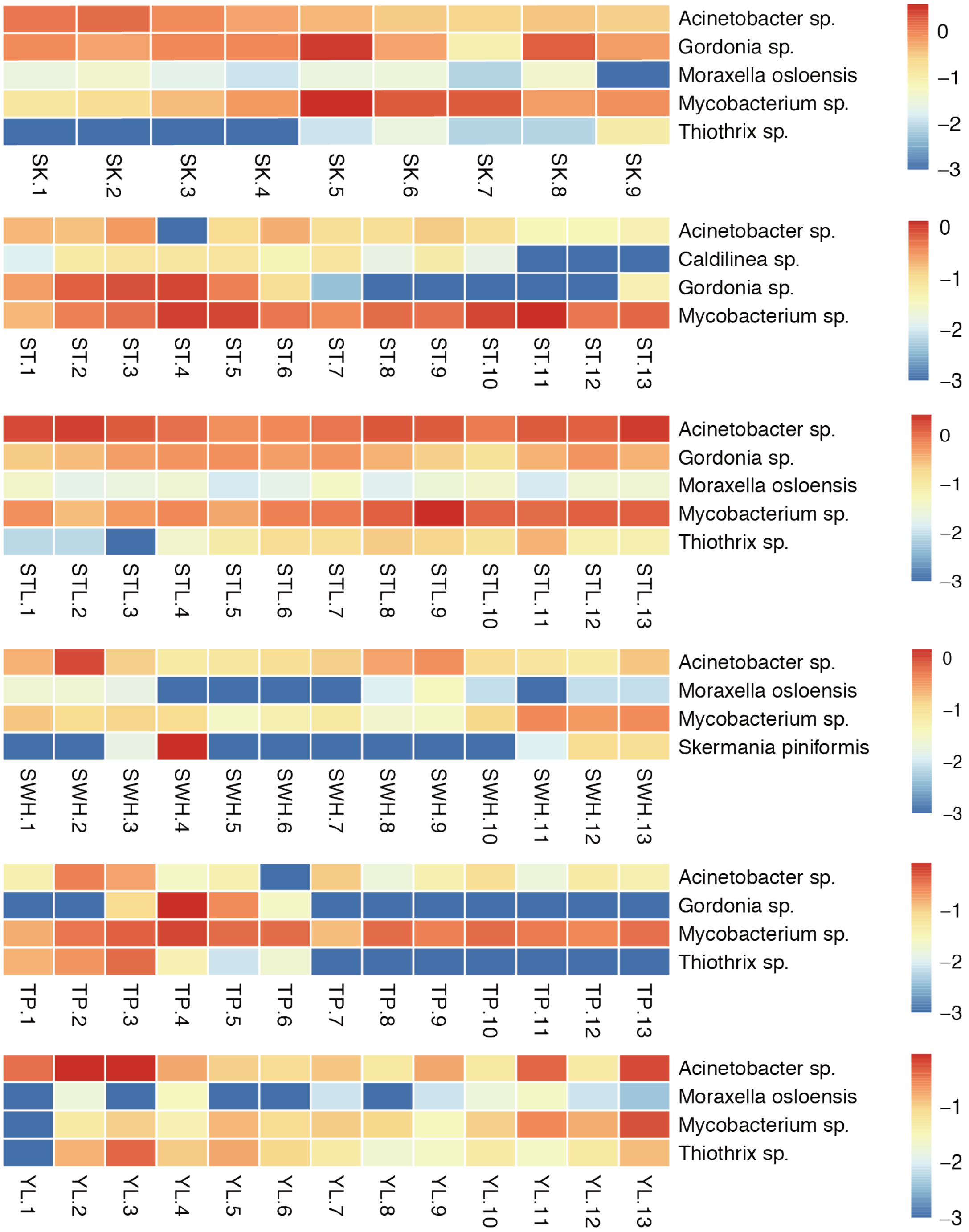

Figure S9 The distribution and abundance of bulking and foaming bacteria in 6 Hong Kong WWTPs. The abundance data were treated by $\log_{10}$. Only the OTUs with average abundance > 0.01% are reserved.

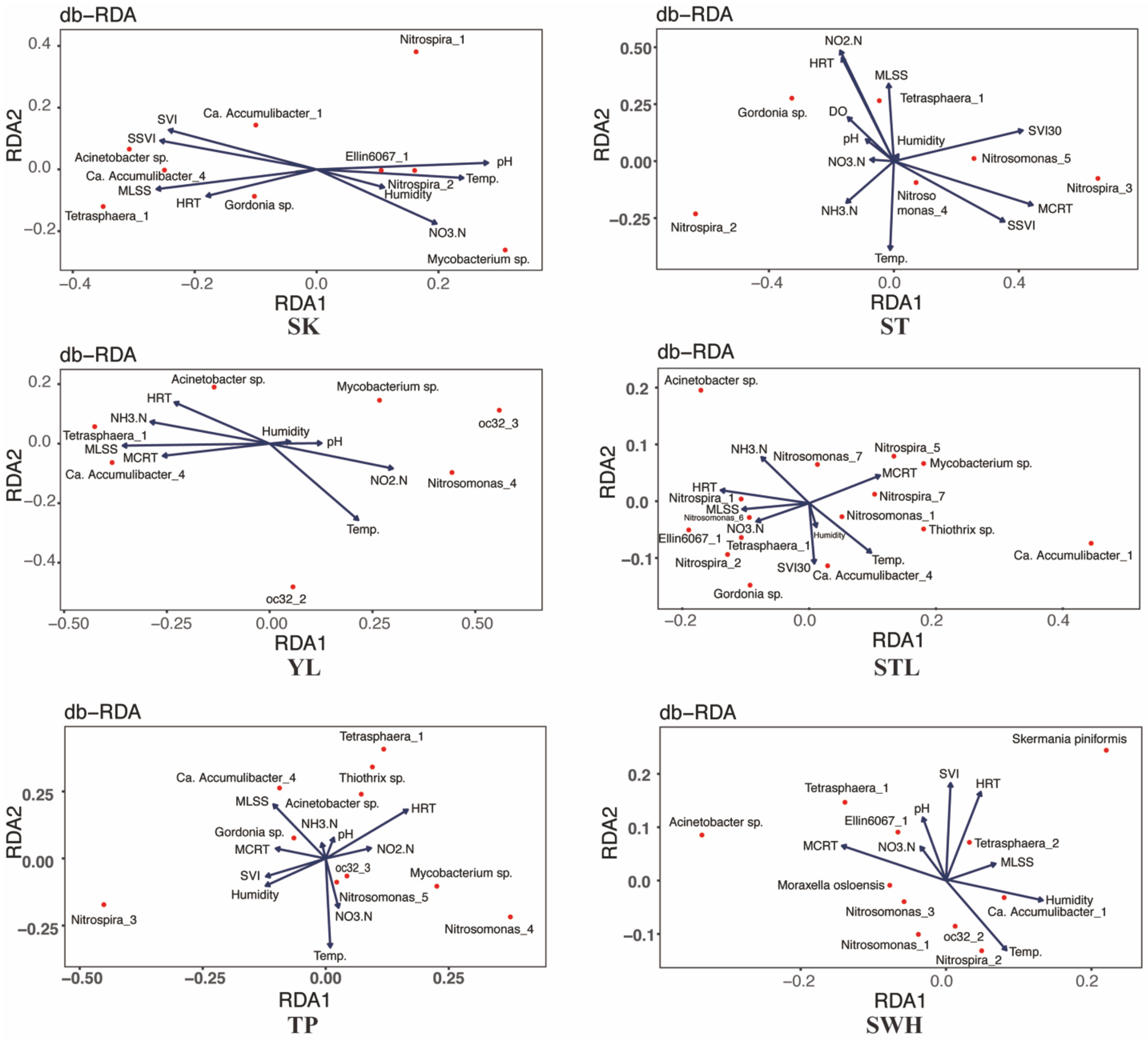

Figure S10 The RDA analyses between functional OTUs and operational parameters of 6 WWTPs. (MCRT: mean cell residence time; MLSS: mixed liquor suspended solid; SVI: sludge volume index; SSVI: stirred specific volume index; Temp.: temperature.)

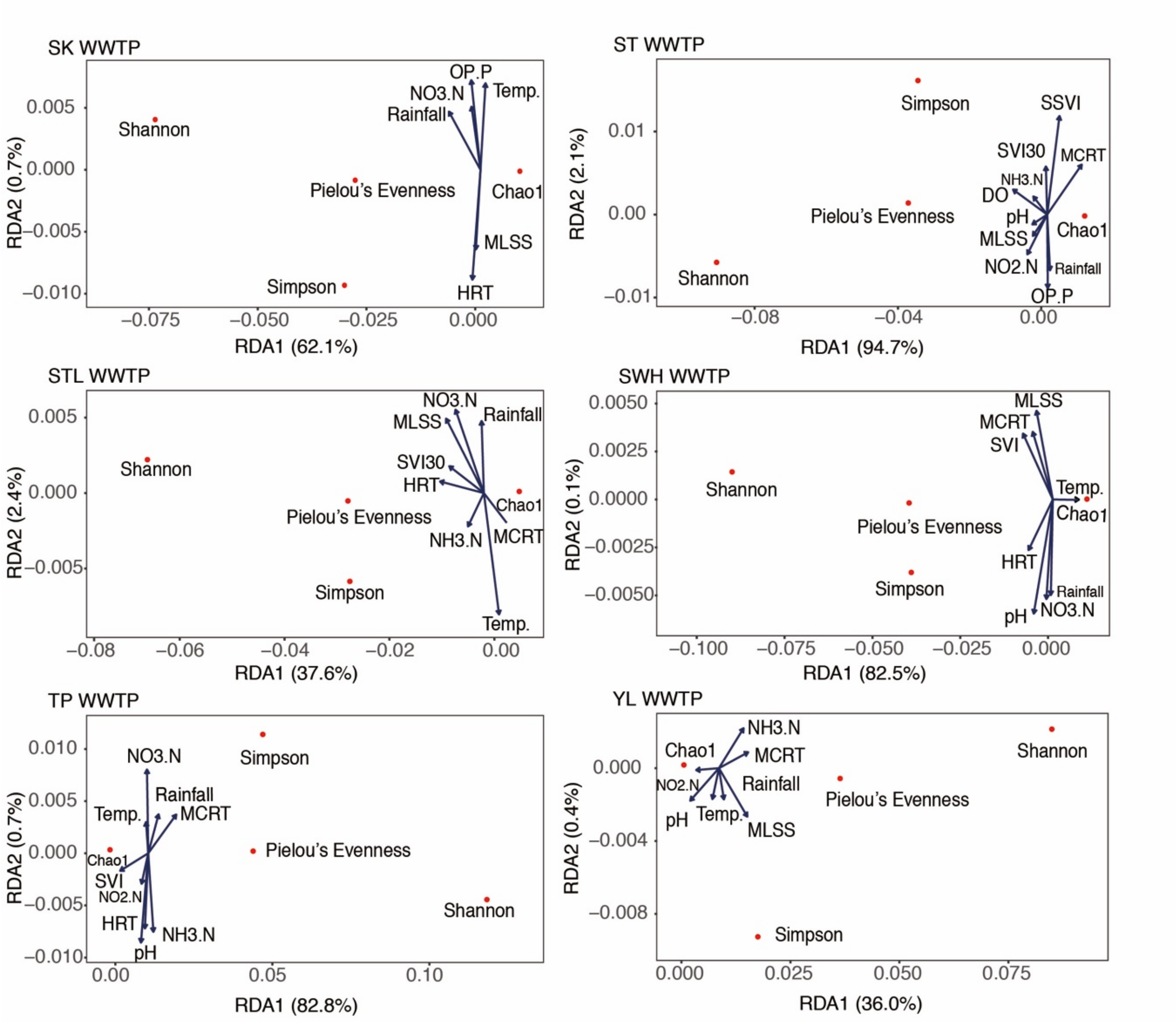

Figure S11 The RDA analyses between operational parameters and α diversity of 6 WWTPs. (MCRT: mean cell residence time; MLSS: mixed liquor suspended solid; SVI: sludge volume index; SSVI: stirred specific volume index; Temp.: temperature.)

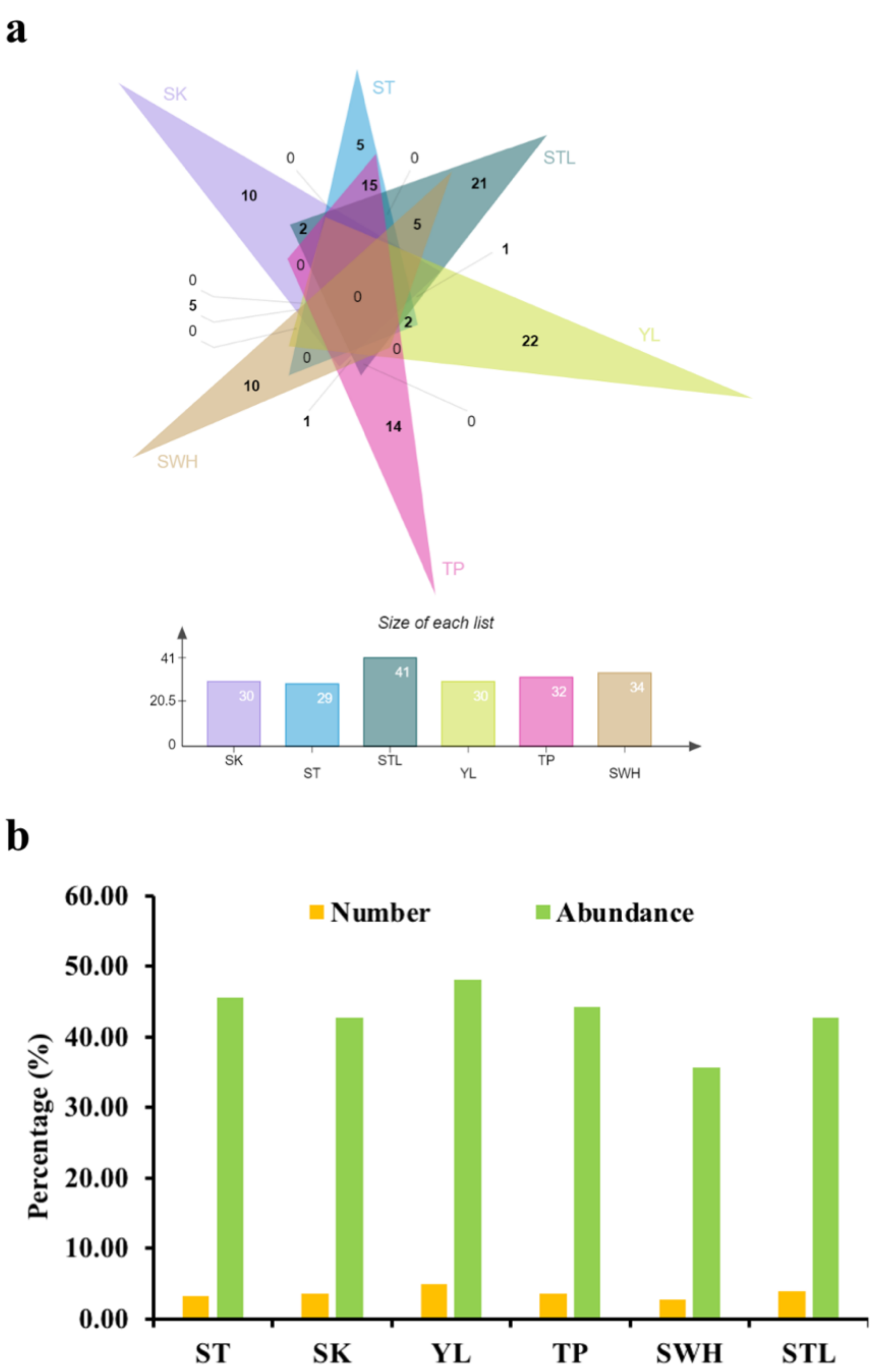

Figure S12 The overlap and distribution of core community for 6 WWTPs. a) Overlap of core community. b) The distribution of number and abundance of core community.

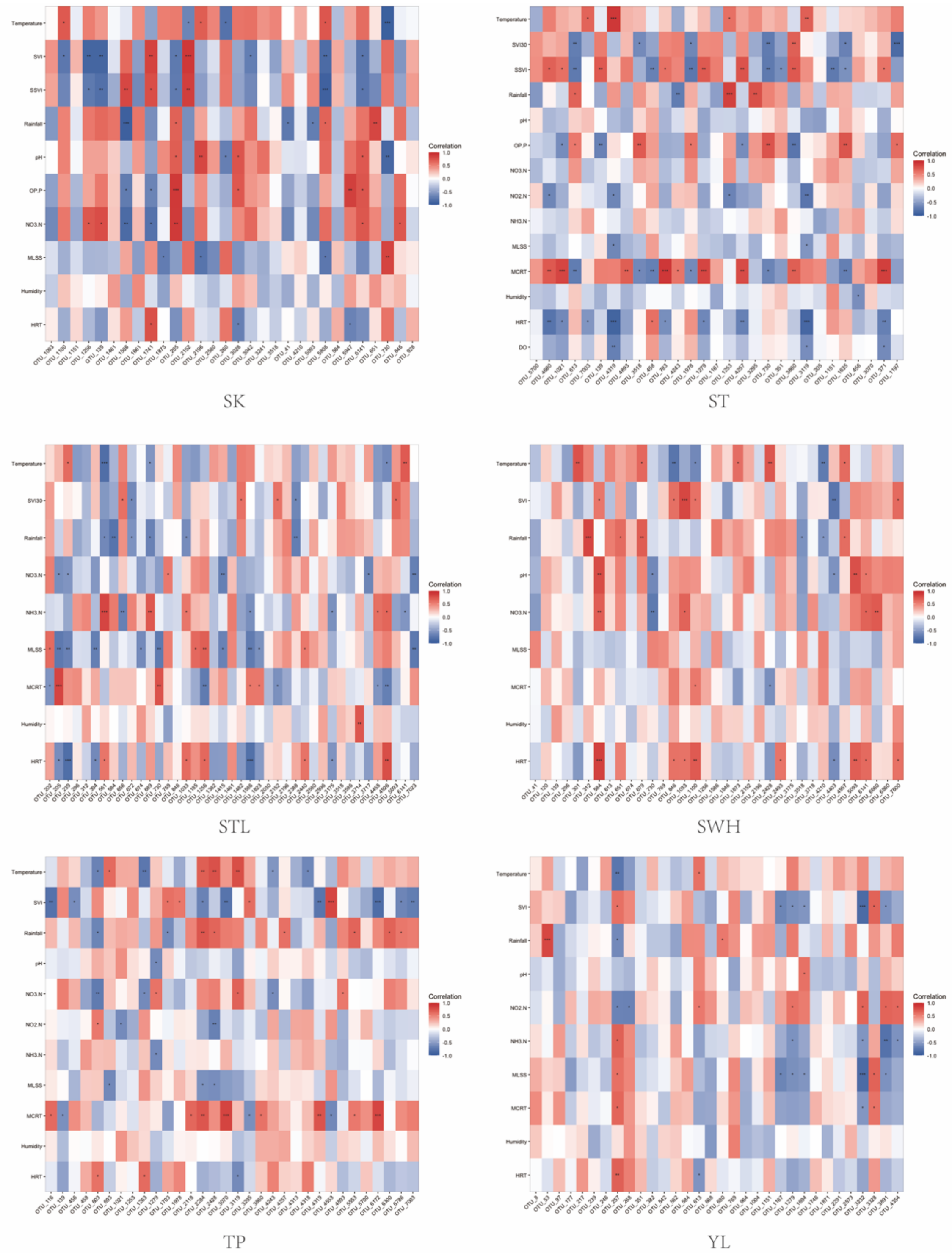

Figure S13 The Spearman results between the core community and operational parameters of 6 WWTPs.
